# Age-stratified and blood-pressure-stratified effects of blood-pressure-lowering pharmacotherapy for the prevention of cardiovascular disease and death: an individual participant-level data meta-analysis

**DOI:** 10.1016/S0140-6736(21)01921-8

**Published:** 2021-09-18

**Authors:** The Blood Pressure Lowering Treatment Trialists' Collaboration, Kazem Rahimi, Zeinab Bidel, Milad Nazarzadeh, Emma Copland, Dexter Canoy, Malgorzata Wamil, Jeannette Majert, Richard McManus, Amanda Adler, Larry Agodoa, Ale Algra, Folkert W Asselbergs, Nigel S Beckett, Eivind Berge, Henry Black, Eric Boersma, Frank P J Brouwers, Morris Brown, Jasper J Brugts, Christopher J Bulpitt, Robert P Byington, William C Cushman, Jeffrey Cutler, Richard B Devereaux, Jamie P Dwyer, Ray Estacio, Robert Fagard, Kim Fox, Tsuguya Fukui, Ajay K Gupta, Rury R Holman, Yutaka Imai, Masao Ishii, Stevo Julius, Yoshihiko Kanno, Sverre E Kjeldsen, John Kostis, Kizuku Kuramoto, Jan Lanke, Edmund Lewis, Julia B Lewis, Michel Lievre, Lars H Lindholm, Stephan Lueders, Stephen MacMahon, Giuseppe Mancia, Masunori Matsuzaki, Maria H Mehlum, Steven Nissen, Hiroshi Ogawa, Toshio Ogihara, Takayoshi Ohkubo, Christopher R Palmer, Anushka Patel, Marc Allan Pfeffer, Bertram Pitt, Neil R Poulter, Hiromi Rakugi, Gianpaolo Reboldi, Christopher Reid, Giuseppe Remuzzi, Piero Ruggenenti, Takao Saruta, Joachim Schrader, Robert Schrier, Peter Sever, Peter Sleight, Jan A Staessen, Hiromichi Suzuki, Lutgarde Thijs, Kenji Ueshima, Seiji Umemoto, Wiek H van Gilst, Paolo Verdecchia, Kristian Wachtell, Paul Whelton, Lindon Wing, Mark Woodward, Yoshiki Yui, Salim Yusuf, Alberto Zanchetti, Zhen-Yu Zhang, Craig Anderson, Colin Baigent, Barry Morton Brenner, Rory Collins, Dick de Zeeuw, Jacobus Lubsen, Ettore Malacco, Bruce Neal, Vlado Perkovic, Anthony Rodgers, Peter Rothwell, Gholamreza Salimi-Khorshidi, Johan Sundström, Fiona Turnbull, Giancarlo Viberti, Jiguang Wang, John Chalmers, Barry R Davis, Carl J Pepine, Koon K Teo

## Abstract

**Background:**

The effects of pharmacological blood-pressure-lowering on cardiovascular outcomes in individuals aged 70 years and older, particularly when blood pressure is not substantially increased, is uncertain. We compared the effects of blood-pressure-lowering treatment on the risk of major cardiovascular events in groups of patients stratified by age and blood pressure at baseline.

**Methods:**

We did a meta-analysis using individual participant-level data from randomised controlled trials of pharmacological blood-pressure-lowering versus placebo or other classes of blood-pressure-lowering medications, or between more versus less intensive treatment strategies, which had at least 1000 persons-years of follow-up in each treatment group. Participants with previous history of heart failure were excluded. Data were obtained from the Blood Pressure Lowering Treatment Triallists' Collaboration. We pooled the data and categorised participants into baseline age groups (<55 years, 55–64 years, 65–74 years, 75–84 years, and ≥85 years) and blood pressure categories (in 10 mm Hg increments from <120 mm Hg to ≥170 mm Hg systolic blood pressure and from <70 mm Hg to ≥110 mm Hg diastolic). We used a fixed effects one-stage approach and applied Cox proportional hazard models, stratified by trial, to analyse the data. The primary outcome was defined as either a composite of fatal or non-fatal stroke, fatal or non-fatal myocardial infarction or ischaemic heart disease, or heart failure causing death or requiring hospital admission.

**Findings:**

We included data from 358 707 participants from 51 randomised clinical trials. The age of participants at randomisation ranged from 21 years to 105 years (median 65 years [IQR 59–75]), with 42 960 (12·0%) participants younger than 55 years, 128 437 (35·8%) aged 55–64 years, 128 506 (35·8%) 65–74 years, 54 016 (15·1%) 75–84 years, and 4788 (1·3%) 85 years and older. The hazard ratios for the risk of major cardiovascular events per 5 mm Hg reduction in systolic blood pressure for each age group were 0·82 (95% CI 0·76–0·88) in individuals younger than 55 years, 0·91 (0·88–0·95) in those aged 55–64 years, 0·91 (0·88–0·95) in those aged 65–74 years, 0·91 (0·87–0·96) in those aged 75–84 years, and 0·99 (0·87–1·12) in those aged 85 years and older (adjusted p_interaction_=0·050). Similar patterns of proportional risk reductions were observed for a 3 mm Hg reduction in diastolic blood pressure. Absolute risk reductions for major cardiovascular events varied by age and were larger in older groups (adjusted p_interaction_=0·024). We did not find evidence for any clinically meaningful heterogeneity of relative treatment effects across different baseline blood pressure categories in any age group.

**Interpretation:**

Pharmacological blood pressure reduction is effective into old age, with no evidence that relative risk reductions for prevention of major cardiovascular events vary by systolic or diastolic blood pressure levels at randomisation, down to less than 120/70 mm Hg. Pharmacological blood pressure reduction should, therefore, be considered an important treatment option regardless of age, with the removal of age-related blood-pressure thresholds from international guidelines.

**Funding:**

British Heart Foundation, National Institute of Health Research Oxford Biomedical Research Centre, Oxford Martin School.

## Introduction

Increased blood pressure is a well known, modifiable risk factor for cardiovascular morbidity and mortality, and antihypertensive medications play an essential cardioprotective role.^1^, ^2^ With ageing populations, one increasingly important uncertainty of the effects of blood-pressure-lowering pharmacotherapy is whether treatment should be initiated in, and continued into, older age (70 years and older), mainly when blood pressure is within the normal range.^3^

**Research in context**

**Evidence before this study**

We searched MEDLINE, the Cochrane Central Register of Controlled Trials, and ClinicalTrials.gov covering the period between Jan 1, 1966, and Sept 1, 2019, with no language restrictions, for randomised controlled trials investigating blood-pressure-lowering drug treatment. We searched MEDLINE using and expanding on the MeSH terms for “hypertension”, “blood pressure”, and “antihypertensive agents”, including possible variations thereof as well as relevant antihypertensive drug classes. We identified several individual randomised controlled trials and meta-analyses with age-stratified effects of blood-pressure-lowering treatment but no reports with concurrent age and blood pressure stratification at the individual level. Additionally, evidence on treatment effects in individuals older than 85 years and with normal or mildly increased blood pressure was scarce.

**Added value of this study**

We gathered individual participant-level data from eligible large-scale trials of blood-pressure-lowering treatment. With access to individual participant-level data from 358 707 randomised participants from 51 trials (with 22 000 participants aged ≥80 years), this study enabled detailed investigation of age-stratified and blood-pressure-stratified effects on major cardiovascular events and death. We found pharmacological blood pressure reduction to be effective across a wide range of ages with no evidence that relative risk reductions for prevention of major cardiovascular events varied by baseline systolic or diastolic blood pressure levels, down to less than 120/70 mm Hg. Although we found evidence for diminishing relative risk reductions with increasing age and limited statistical power for detection of an effect in the oldest age group in isolation (90 years at the end of the study), absolute risk reductions did not follow the same pattern and appeared to be even larger in the older age groups. Stratified effects on all-cause death followed a similar pattern, with no evidence to suggest treatment increases mortality in any age group.

**Implications of all the available evidence**

This detailed study of age-stratified and blood-pressure-stratified effect of antihypertensive medication provides compelling evidence for the effectiveness of pharmacological blood pressure reduction into old age irrespective of baseline systolic or diastolic blood pressure. These findings challenge the common approach of withholding antihypertensive treatment for older adults, in particular when their blood pressure is not highly abnormal. Treatment should, therefore, be considered an important option regardless of age with removal of age-related blood-pressure thresholds from international guidelines.

Epidemiological studies have suggested that increased blood pressure is a major risk factor for cardiovascular events across different age categories and over a wide range of blood pressure.^4^, ^5^, ^6^, ^7^ Although these studies have found some attenuation in relative risks with increasing age, older patients might still gain as much as, if not more than, younger individuals from blood-pressure-lowering treatment because the absolute cardiovascular event rates increase with age.^5^ However, other studies have reported an increased risk of cardiovascular events and death in older patients with lower blood pressure compared with those with higher blood pressure.^8^, ^9^, ^10^, ^11^ Some have even suggested a rapid decline in blood pressure in the years preceding death, raising doubts about the value of blood-pressure-lowering treatment in older people.^12^

Thus far, robust evidence from randomised controlled trials (RCTs) has been lacking, in part because of the under-representation of older individuals in clinical trials. To date, the Hypertension in the Very Elderly Trial (HYVET) is the only large-scale trial that has exclusively recruited patients aged 80 years and older.^13^ Although this study found 30% reductions in risk of stroke and 23% reductions in risk of cardiovascular death, its 3845 participants were selected on the basis of having very high blood pressure (systolic blood pressure 160 mm Hg or higher) at baseline. Several other randomised trials and their meta-analyses have also investigated the effects of blood pressure reduction by age.^14^, ^15^, ^16^ Although these studies found no evidence of heterogeneity of effects by age, individual trials have had insufficient statistical power to investigate this question in depth. Previous meta-analyses were also mainly based on broad age categories (eg, <65 years *vs* ≥65 years) and could not investigate effects based on narrower age groups and by other important characteristics such as baseline blood pressure.^15^ This uncertainty is evident in the conflicting clinical guideline recommendations for treatment according to age (appendix p 2).^17^, ^18^, ^19^

The third cycle of the Blood Pressure Lowering Treatment Trialists' Collaboration (BPLTTC) had access to individual participant-level data of over 350 000 randomly assigned patients with 22 000 patients aged 80 years and older.^20^ These data offered an unprecedented opportunity to do an individual participant-level data meta-analysis of RCTs to investigate the stratified effects of pharmacological blood-pressure-lowering treatment on the risk of major cardiovascular events and death across age, systolic, and diastolic blood pressure categories at baseline.

## Methods

### Search strategy and selection criteria

In this meta-analysis of individual participant-level data, we used the resources provided by the BPLTTC. The BPLTTC is a collaboration of the principal investigators of major clinical trials of pharmacological blood-pressure-lowering treatment. The collaboration is coordinated by the University of Oxford (Oxford, UK) and, based on the last update, includes information from 52 randomised trials.^20^ Details of the BPLTTC design have been reported elsewhere.^2^, ^20^, ^21^ The initial inclusion criteria of the BPLTTC were RCTs of pharmacological blood-pressure-lowering treatment with at least 1000 persons-years of follow-up in each randomly allocated group. In this analysis, we included trials that provided data for outcomes, including type and timing of events, as well as age and baseline blood pressure measurements. Participants with previous history of heart failure were excluded. We searched MEDLINE using and expanding on the MeSH terms for “hypertension”, “blood pressure”, and “antihypertensive agents” including possible variations thereof as well as relevant antihypertensive drug classes. Details of the selection and identification of eligible trials have been described previously.^20^ The risk of bias was assessed using the Cochrane risk-of-bias tool, which has been reported elsewhere.^2^

Ethics approval for this phase was obtained from the Oxford Tropical Research Ethics Committee (OxTREC reference 545–14), and the analysis plan was approved by the BPLTTC steering committee and collaborators before releasing the data for analysis.

### Outcomes, randomised groups, and stratification variables

The primary outcome was defined as either a composite of fatal or non-fatal stroke, fatal or non-fatal myocardial infarction or ischaemic heart disease, or heart failure causing death or requiring hospital admission. The secondary outcomes were all-cause death and each component of the primary outcome. For each trial, randomised groups were classified into the two groups of “intervention” and “comparator”. For placebo-controlled trials, the placebo group was considered as the comparator and the active group as the intervention. For trials comparing different drug classes, the group in which the blood pressure reduction was greater was considered as intervention and the other treatment groups as comparator. Trials that compared more intense versus less intense strategies were classified as the intervention versus comparator groups. Detailed information about the comparison groups, trial design, patient characteristics, and level of blood pressure reduction for each trial have been reported elsewhere.^2^, ^20^, ^21^

To identify age-specific effects, we categorised participants into five groups that were based on their age at baseline (younger than 55 years, 55–64 years, 65–74 years, 75–84 years, and 85 years or older). To examine the risk reduction across blood pressure categories, we stratified the participants into seven categories of baseline systolic blood pressure (<120 mm Hg, 120–129 mm Hg, 130–139 mm Hg, 140–149 mm Hg, 150–159 mm Hg, 160–169 mm Hg, and ≥170 mm Hg) and six categories of baseline diastolic blood pressure (<70 mm Hg, 70–79 mm Hg, 80–89 mm Hg, 90–99 mm Hg, 100–109 mm Hg, and ≥110 mm Hg).

### Data analysis

We did a one-stage, individual participant-level data, meta-analysis using stratified Cox proportional hazard models, with fixed treatment effects, and participants as the units of analysis.^2^, ^22^ The model was stratified by baseline hazard functions for each trial to satisfy the proportional hazards assumption.^23^ We did an intention-to-treat analysis that was based on the groups to which each participant had initially been assigned (intervention *vs* comparator). Patients entered the analysis at the date of the randomisation and were followed up until the earliest occurrence of the outcome of interest, death, or end of the trial. Mean systolic blood pressure reduction between randomised groups, excluding the first 12 months, among all trials that aimed at achieving a difference in blood pressure, was 6·3 mm Hg (95% CI 6·1–6·4) and the average diastolic blood pressure reduction was 3 mm Hg (2·9–3·0).^21^ Therefore, we standardised the effect sizes for each 5 mm Hg reduction in systolic and 3 mm Hg for diastolic blood pressure reduction. The method used for this standardisation and detailed description of the statistical analyses have been published elsewhere.^2^

We plotted cumulative incidence curves by treatment allocation and age categories. Hazard ratios (HRs) and their 95% CIs were presented using forest plots with standardisation by 5 mm Hg reductions in systolic blood pressure and 3 mm Hg reductions in diastolic blood pressure. To test whether treatment effects varied across prespecified subgroups of age categories and systolic and diastolic blood pressure at baseline, we used likelihood-ratio tests for interactions. Likelihood-ratio tests compared models with and without interactions between treatment effect and age or blood pressure categories. The calculated p_interaction_ was adjusted for multiple testing using Hommel's method to avoid chance finding.^24^, ^25^ As a sensitivity analysis, we estimated the unstandardised effect, which did not consider weighting treatment effects by the achieved blood pressure reduction for each trial. These analyses were prespecified and followed the study protocol. We also did a sensitivity analysis investigating the unstandardised age-stratified effects and additionally stratified by the three types of trial designs. We further calculated the absolute risk reductions using a Poisson regression model with identity link for each stratum to investigate the heterogeneity of treatment effects on an absolute scale. For this analysis, the absolute risk difference would reflect the mean blood pressure reduction across all trials contributing data for each category. Analyses were done using R (version 3.3).

### Role of the funding source

The funder of the study had no role in study design, data collection, data analysis, data interpretation, or writing of the report.

## Results

Of the 52 randomised trials included in the BPLTTC, we excluded one trial because it did not report the outcome of interest. Therefore, 51 trials comprising 358 707 participants were included in the analysis (appendix p 2). We found no reports of heart failure outcome in six (12%) trials, no cardiovascular death outcomes in five (10%) trials, and no stroke and ischaemic heart disease outcomes in one (2%) trial (appendix p 2). Of the included participants, 42 960 (12·0%) were aged 55 years or younger, 128 437 (35·8%) aged 55–64 years, 128 506 (35·8%) aged 65–74 years, 54 016 (15·1%) aged 75–84 years, and 4788 (1·3%) were aged 85 years or older (range 21–105 years). The highest median follow-up time was in those aged 55 years or younger (4·5 years [IQR 3·1]) and the lowest median follow-up was in those aged 85 years and older (2·8 years [2·3]). Compared with men, the percentage of women was higher in older age groups and lower in younger age groups (table). The prevalence of peripheral vascular disease, atrial fibrillation, and cerebrovascular disease at baseline were greater in the older age groups (table). Mean systolic blood pressure at baseline was higher and diastolic blood pressure was lower in older age groups (table). Detailed characteristics of participants stratified by age categories are presented in the table.

**Table:** Baseline characteristics of participants by age categories at baseline

		**<55 years (n=42960)**	**55–64 years (n=128 437)**	**65–74 years (n=128 506)**	**75–84 years (n=54 016)**	**≥85 years (n=4788)**
Sex						
	Female	14 957 (34·8%)	49 785 (38·8%)	53 696 (41·8%)	27 835 (51·5%)	2938 (61·4%)
	Male	28 003 (65·2%)	78 652 (61·2%)	74 810 (58·2%)	26 181 (48·5%)	1850 (38·6%)
Systolic blood pressure (mm Hg)		150 (20·7)	150 (20·4)	153 (21·2)	158 (22·3)	157 (20·5)
Diastolic blood pressure (mm Hg)		95 (12·4)	88 (11·9)	86 (11·8)	84 (11·9)	82 (12·1)
Categories of systolic blood pressure (mm Hg)						
	<120	2384 (5·6%)	7151 (5·6%)	5415 (4·2%)	1646 (3·1%)	121 (2·5%)
	120–129	3864 (9·1%)	12 537 (9·8%)	10 137 (7·9%)	3285 (6·1%)	305 (6·4%)
	130–139	6164 (14·5%)	19 212 (15·1%)	16 467 (12·9%)	5753 (10·7%)	510 (10·7%)
	140–149	8535 (20·1%)	25 058 (19·6%)	23 260 (18·2%)	8524 (15·8%)	738 (15·4%)
	150–159	7366 (17·3%)	22 798 (17·9%)	21936 (17·1%)	8140 (15·1%)	644 (13·5%)
	160–169	6738 (15·9%)	19 539 (15·3%)	22 846 (17·9%)	10794 (20·0%)	1012 (21·1%)
	≥170	7433 (17·5%)	21 230 (16·6%)	27 888 (21·8%)	15 821 (29·3%)	1458 (30·5%)
Categories of diastolic blood pressure (mm Hg)						
	<70	945 (2·2%)	6108 (4·8%)	9778 (7·6%)	5882 (10·9%)	708 (14·8%)
	70–79	3270 (7·7%)	20 025 (15·7%)	25 662 (20·1%)	12243 (22·7%)	1091 (22·8%)
	80–89	7527 (17·7%)	38 509 (30·2%)	42 986 (33·6%)	17 808 (33·0%)	1378 (28·8%)
	90–99	13 731 (32·3%)	37 490 (29·4%)	32 556 (25·4%)	12 737 (23·6%)	1299 (27·2%)
	100–109	12 384 (29·1%)	19 969 (15·7%)	13485 (10·5%)	4233 (7·8%)	278 (5·8%)
	≥110	4628 (10·9%)	5425 (4·3%)	3472 (2·7%)	1056 (2·0%)	30 (0·6%)
Body mass index (kg/m^2^)		28·3 (5·0)	28·7 (5·3)	27·8 (10·0)	26·4 (4·8)	25·2 (4·0)
Comorbidity						
	Peripheral vascular disease	763 (5·1%)	4208 (9·0%)	5432 (10·6%)	2287 (11·6%)	207 (14·0%)
	Atrial fibrillation	550 (1·3%)	2213 (1·7%)	4356 (3·4%)	3058 (5·7%)	308 (6·4%)
	Diabetes*	7257 (16·9%)	40 686 (31·7%)	41 269 (32·1%)	13 199 (24·4%)	807 (16·9%)
	Chronic kidney disease	4562 (16·4%)	7893 (16·1%)	7725 (16·2%)	3634 (19·1%)	247 (16·0%)
	Cerebrovascular disease	3780 (9·5%)	16 946 (17·2%)	19 700 (19·6%)	9484 (21·0%)	737 (19·1%)
	Ischaemic heart disease	13 035 (30·8%)	42 689 (33·6%)	45 813 (35·9%)	17 045 (31·6%)	1414 (29·5%)
Previous use of non-study medications						
	ACEIs	2129 (18·1%)	17 478 (33·8%)	19 862 (34·0%)	8206 (31·6%)	674 (31·8%)
	ARBs	390 (4·1%)	1961 (6·0%)	3805 (9·5%)	2353 (13·5%)	65 (8·1%)
	Calcium-channel blockers	3960 (27·7%)	18 172 (30·0%)	23 400 (34·2%)	9888 (33·4%)	610 (28·0%)
	Diuretics	1696 (11·9%)	10 563 (18·4%)	14 451 (22·5%)	7032 (25·1%)	672 (30·9%)
	β-blockers	5565 (39·0%)	22 382 (36·9%)	23 597 (34·5%)	8007 (27·0%)	381 (17·5%)
	α-blockers	321 (4·9%)	1299 (3·2%)	2118 (4·4%)	1061 (4·8%)	53 (5·3%)
	Anti-platelet medications	1319 (27·7%)	19 823 (47·1%)	21 431 (45·3%)	7982 (36·3%)	476 (25·3%)
	Anticoagulants medications	304 (6·0%)	1519 (5·0%)	2893 (8·0%)	1763 (13·7%)	91 (13·3%)
	Lipid-lowering medications	4459 (35·1%)	21 546 (41·5%)	21 674 (37·5%)	6646 (27·8%)	154 (8·9%)
Follow-up (years)		4·5 (3·1)	4·4 (2·0)	4·1 (1·9)	3·7 (2·2)	2·8 (2·3)

Data are n (%) or mean (SD). ACEI=angiotensin-converting enzyme inhibitor. ARB=angiotensin-receptor blocker.

* Of any type.

**Figure 1:**
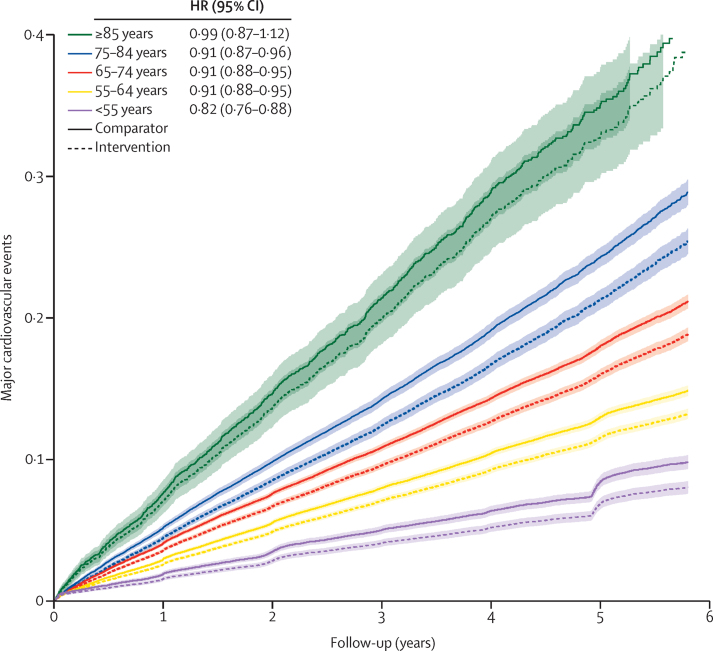
Rate of major cardiovascular events per 5 mm Hg reduction in systolic blood pressure, stratified by treatment allocation and age categories at baseline Major cardiovascular events, defined as a composite of fatal or non-fatal stroke, fatal or non-fatal myocardial infarction or ischaemic heart disease, or heart failure causing death or requiring hospital admission. The shaded area represents the 95% CIs.

**Figure 2:**
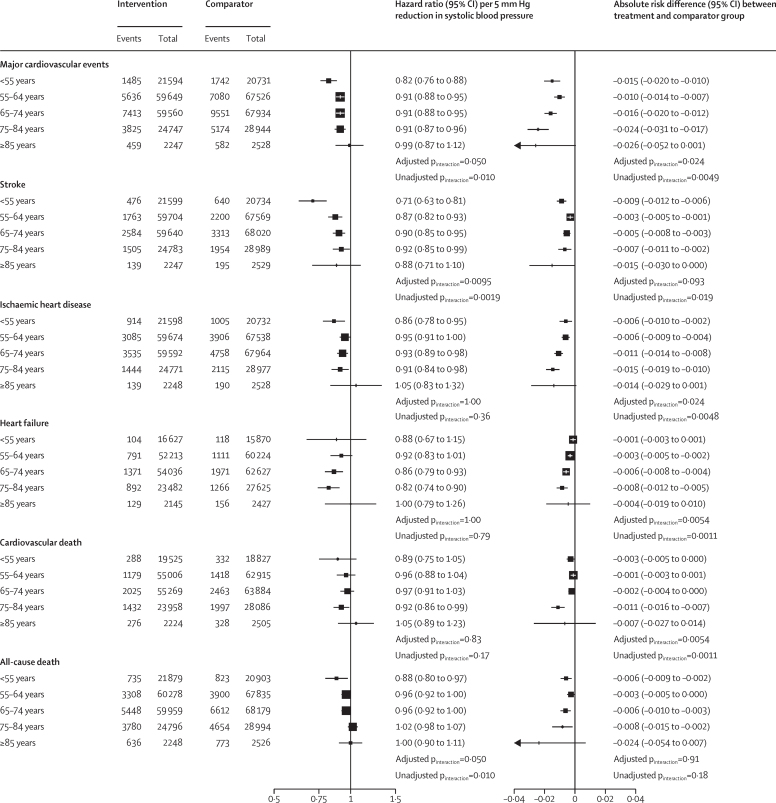
Age-stratified relative risk and absolute risk difference of systolic blood pressure reduction on primary and secondary outcomes Relative risk reductions are presented with hazard ratios and 95% CIs per 5 mm Hg reduction in systolic blood pressure, separately for each outcome. The absolute risk difference reflects the mean blood pressure reduction for each age category. Adjusted p_interaction_ was adjusted for multiple testing using Hommel's method. Unadjusted p_interaction_ was unadjusted for multiple testing.

The cumulative incidence for the primary outcome stratified by age categories at baseline and treatment allocation showed an increasing incidence by increasing age (figure 1). In all age groups, event rates were lower in the intervention than in the comparator group (figure 1). However, the confidence limit was widest in the group of individuals aged 85 years or older at baseline, reflecting the smaller number of participants and events in this group (figure 1). The age-stratified relative and absolute risk reductions for the primary and secondary outcomes are shown in figure 2. For the primary outcome of major cardiovascular events, we found evidence for heterogeneous treatment effects by age, with a pattern consistent with a greater relative risk reduction in the youngest age group and smaller effects with wider CIs in those aged 85 or older at baseline (adjusted p_interaction_=0·050). A pharmacological reduction of 5 mm Hg in systolic blood pressure lowered the risk of major cardiovascular events in participants aged 55 years or younger (HR 0·82 [95% CI 0·76–0·88]), those aged 55–64 years (0·91 [0·88–0·95]), those aged 65–74 years (0·91 [0·88–0·95]), and those aged 75–84 years (0·91 [0·87–0·96]; figure 2). The relative treatment effect in participants aged 85 years or older in isolation was not significant (0·99 [0·87–1·12]; figure 2). However, because of the higher event rate in the older groups, we observed a somewhat higher absolute reduction in the risk of major cardiovascular events in the older groups (adjusted p_interaction_=0·024; figure 2). Broadly similar patterns of absolute and relative risk reductions were observed for the secondary outcomes of stroke, ischaemic heart disease, heart failure, cardiovascular death, and all-cause death (figure 2). However, because of the small numbers of events, the effect estimates were less precise for some of these outcomes compared with major cardiovascular events (figure 2). Relative risk reductions for a reduction of 3 mm Hg in diastolic blood pressure are presented in the appendix (p 8) and were consistent with those for a reduction of 5 mm Hg in systolic blood pressure.

**Figure 3:**
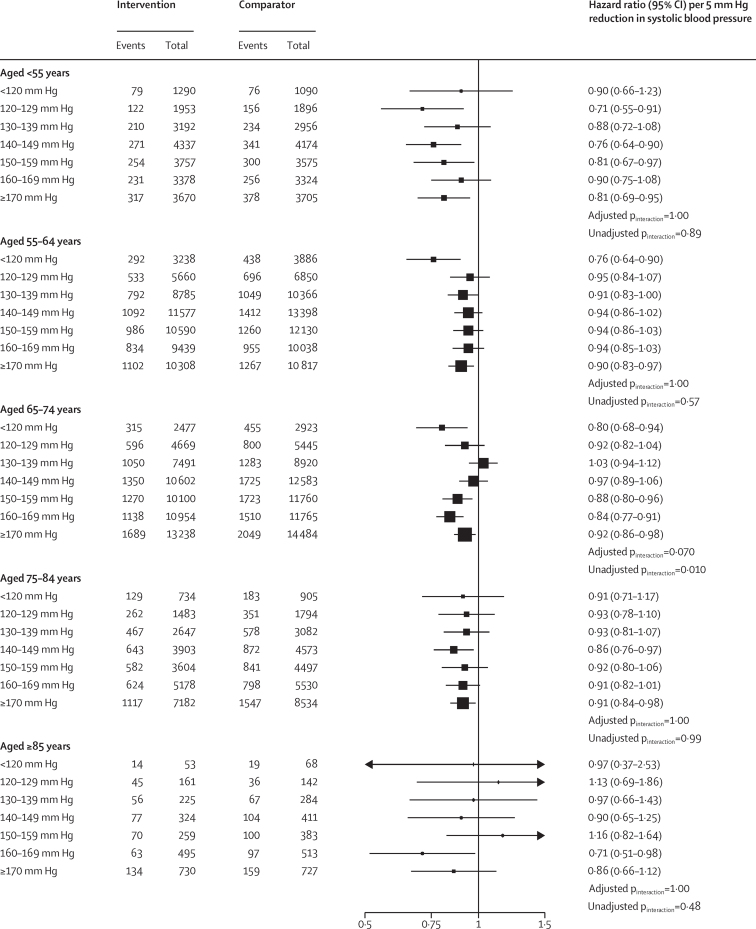
Age-specific relative effects of blood-pressure-lowering treatment on major cardiovascular events, by systolic blood pressure categories at baseline Forest plot shows the hazard ratios and 95% CIs per 5 mm Hg reduction in systolic blood pressure. Adjusted p_interaction_ was adjusted for multiple testing using Hommel's method. Unadjusted p_interaction_ was unadjusted for multiple testing.

**Figure 4:**
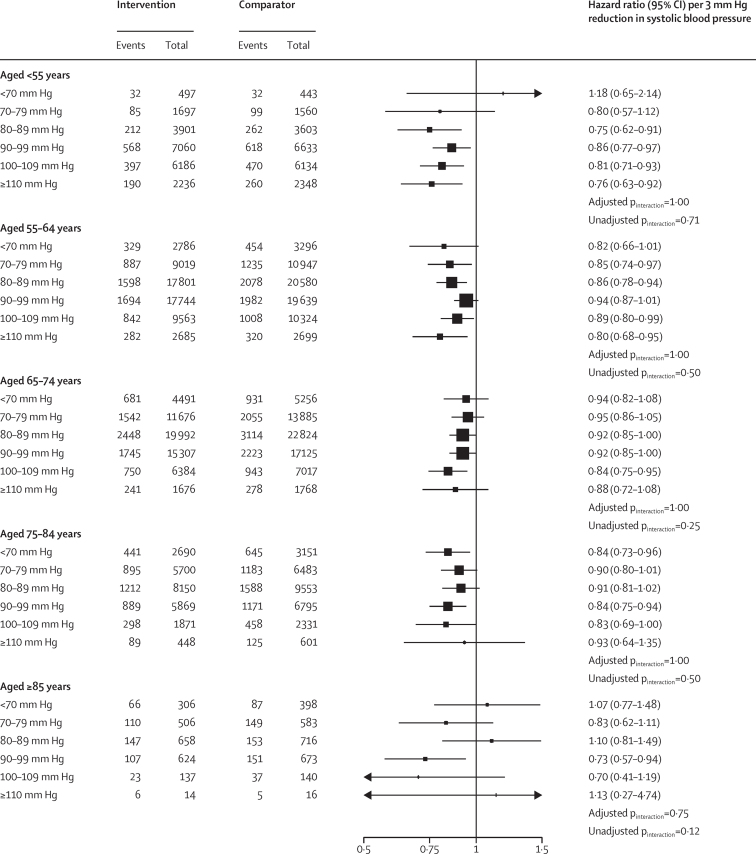
Relative effects of blood-pressure-lowering treatment on major cardiovascular events, by diastolic blood pressure categories at baseline Forest plot shows the hazard ratios and 95% CIs per 3 mm Hg reduction in diastolic blood pressure. Adjusted p_interaction_ was adjusted for multiple testing using Hommel's method. Unadjusted p_interaction_ was unadjusted for multiple testing.

To assess whether the average treatment effects in each age group varied by baseline blood pressure of individuals, we further stratified individuals in each age group into seven prespecified subgroups of systolic blood pressure. This analysis showed no evidence of any heterogeneous treatment effect by categories of systolic blood pressure at baseline on the risk of major cardiovascular events in any of the age groups (all adjusted p_interaction_>0·070; figure 3). In particular, while the CIs of effect estimates were less precise for those aged 85 years compared with other age groups, we found no evidence to suggest that the overall weaker effects in this age group were masking heterogenous treatment effects by baseline blood pressure (adjusted p_interaction_=1·00; figure 3). We also found no clear pattern of increasing proportional effects among individuals with higher baseline systolic blood pressure on the risk of major cardiovascular events (figure 3). Similarly, the effects of diastolic blood pressure reduction stratified by baseline diastolic blood pressure categories and age groups on the risk of major cardiovascular events showed no heterogeneity of treatment by diastolic blood pressure at baseline (all adjusted p_interaction_>0·75; figure 4). Analyses of the treatment effects on the risk of all-cause mortality stratified by age and blood pressure were broadly consistent with the results of major cardiovascular outcomes, showing no evidence of diminishing relative effects in lower systolic (appendix p 9) or diastolic blood pressure categories (appendix p 10).

In a sensitivity analysis, we repeated the analysis without standardisation for blood pressure reduction at the trial level and found that the findings were broadly similar to the main results, both overall (appendix p 11) and when stratified by the three types of trial designs included (appendix p 7).

## Discussion

This individual-level meta-analysis showed that pharmacological blood pressure reduction is effective across a wide range of ages with no evidence to suggest that relative risk reductions for prevention of major cardiovascular events vary by baseline systolic or diastolic blood pressure levels, down to less than 120/70 mm Hg.Although we found evidence for diminishing relative risk reductions with increasing age, absolute risk reductions did not follow the same pattern and appeared to be largest in the oldest age groups. Stratified effects on all-cause death followed a similar pattern, with no evidence to suggest that treatment increased deaths in any age group. With only about 1000 major cardiovascular events accrued over a median follow-up duration of 2·8 years in the group of participants aged 85 years or older at randomisation, the treatment effects in this highest age category in isolation were uncertain.

Representative national surveys in England have shown that systolic blood pressure increases continuously with age.^26^ This pattern has traditionally been considered as a natural process of ageing that is essential for maintenance of coronary and cerebral perfusion. However, the observation that in remote rural populations from non-industrialised regions, blood pressure does not increase with age,^27^, ^28^ as well as epidemiological studies showing strong associations between increased blood pressure and cardiovascular disease across all age groups^4^, ^6^, ^7^ and down to a systolic blood pressure of 90 mm Hg in healthy adults,^29^ have gradually shifted the perception that a higher blood pressure in older individuals is inevitable and physiologically necessary. However, randomised evidence on the effect of pharmacological blood-pressure-lowering on cardiovascular outcomes in individuals older than 80 years across a wide range of blood pressure levels has been insufficient, leading to conflicting guideline recommendations across the world. To our knowledge, the 2017 American College of Cardiology and American Heart Association guidelines are an exception in not making an age distinction for their treatment recommendation.^18^ By contrast, the European Society of Cardiology and European Society of Hypertension set the threshold for consideration of drug treatment in those aged 60–79 years at 140/90 mm Hg or greater and in those aged 80 years or older at 160/90 mm Hg or greater.^17^ Similarly, the 2019 National Institute for Health and Care Excellence guidelines for England do not recommend treatment in adults older than 80 years if their blood pressure is lower than 150/90 mm Hg.^19^ The 2017 American College of Physicians and American Academy of Family Physicians guidelines even consider treatment in patients older than 60 years as only indicated when systolic blood pressure is greater than 150 mm Hg.^30^ The more recent guidelines by the International Society of Hypertension also make a distinction by age and recommend a target of less than 140/90 mm Hg for those aged 65 years and older.^31^

The findings from our study close the gap in evidence for age-specific treatment effects on major cardiovascular outcomes. With access to individual-level data including detailed systolic and diastolic blood pressure measurements from 54 016 randomised participants aged 75–84 and 4788 participants aged 85 years or older, we were able to investigate the effects of treatment to greater depth than before and, importantly, with simultaneous stratification by systolic or diastolic blood pressure down to less than 120/70 mm Hg at randomisation. For our primary and secondary outcomes, we found no strong evidence of heterogeneity of relative effects across a wide range of systolic or diastolic blood pressure categories. These findings are in line with a report^2^ by the BPLTTC that had shown consistent effects for primary and secondary prevention of cardiovascular disease with no evidence of diminishing effects when systolic blood pressure was normal or mildly increased. The present study extends those earlier findings to diastolic blood pressure of less than 70 mm Hg and challenges the differential treatment recommendation by age and blood pressure for the prevention of major cardiovascular events.

The second main finding of this study was the observation that relative risk reductions appeared to diminish with increasing age. The reasons behind this observation are not entirely clear and could be of statistical or biological nature. With a p_interaction_ of 0·05 for the primary outcome and the absence of any meaningful interaction by age for ischaemic heart disease and heart failure, a chance finding cannot be ruled out. Indeed, an alternative interpretation of our age-stratified results could be that relative risk reductions for most participants included in the analyses are consistent. On the other hand, statistical tests for interaction are notoriously conservative and our results in the context of large-scale epidemiological studies,^4^, ^6^ which have also shown a pattern of diminishing relative effects with increasing age, invites consideration of different explanations. For instance, the shorter treatment duration in older participant groups and their longer life-time exposure to increased blood pressure might limit the reversibility of the vascular effects of treatment over a short period of time. Of note, the average treatment duration in the oldest age group was only about half of that in the youngest age group. Furthermore, younger people are less likely to present with multiple risk factors for cardiovascular disease than older people, and this difference could also explain the stronger relative contribution of a single risk-factor modification in younger age compared with older age assuming that overt risk-factor clustering for cardiovascular disease diminishes relative risks while increasing absolute risks.

Regardless of whether the heterogeneity of relative treatment effects by age are meaningful or spurious, the absolute risk reductions afforded by the treatment more convincingly increased with age because of the substantially increasing risk of vascular events by age. The strength of RCTs lies in their ability to provide unbiased estimates of relative treatment effects that are typically generalisable across time and place. However, estimates of absolute risks are less generalisable because trial participants are rarely representative of populations to whom the results are to be applied. Therefore, we caution against overinterpretation of our results by assuming that the absolute risk reductions reported are fixed and directly applicable to decision making. More appropriately, such estimates are to be derived from the combination of proportional effects in our study and absolute risks taken from contemporary patient registries.^32^ Thus, our analyses of absolute risk differences are only useful for internal comparisons of effect sizes across strata. To this end, the observation of increasing absolute risk reductions in older participant groups should help overcome the clinical inertia and the common inverse care law to which many older individuals are subjected.^33^

Our analyses focused on the effects of a fixed degree of blood pressure reduction on future risk of major cardiovascular events, including its components of stroke, heart failure, ischaemic heart disease, and cardiovascular death. We also report the effects on all-cause death, which might be of particular interest to guide decision making for blood-pressure-lowering pharmacotherapy in older individuals. We found that the proportional risk reduction for all-cause death is marginally larger in younger than in older age, with no obvious effect in people older than 75 years. Generally, treatment effects on all-cause death from targeted interventions in RCTs are to be interpreted with caution because of their sensitivity to varying fractions of outcomes that are amenable to treatment and those that are unlikely to be affected by them. For instance, in another BPLTTC report, we have shown that blood-pressure-lowering pharmacological therapy has no material effect on cancer risk in those receiving active intervention.^34^ But, if cancer death rate is substantially increased in one subgroup, then one would expect a dilution of proportional treatment effects in comparison with another subgroup that has a higher fraction of cardiovascular death, despite consistent effects on cardiovascular events. With these considerations in mind, the lack of excess mortality risk in older groups suggests that harmful fatal effects of the treatment are unlikely in any age group.

Health-related quality of life and prevention of harms might be of equal or even greater importance to older people than prevention of fatal or non-fatal cardiovascular events. However, to our knowledge, no randomised comparisons exist to suggest that a fixed level of blood pressure reduction in older individuals causes more harm than benefit in older people. For instance, in a subgroup analysis of the Systolic Blood Pressure Intervention Trial (SPRINT), the total serious adverse event rates were similar in the two study groups.^35^ Several other age-stratified analyses of RCTs and their meta-analyses have also shown no worsening in functional status, physical wellbeing, or quality of life in older people.^36^ Concerns about worsening cognitive function in older people and low blood pressure have been raised in some observational studies but are likely to be due to reverse causation^37^ and have not been substantiated in a randomised trial thus far.^38^ Planned BPLTTC projects are investigating some of those questions in greater detail.^20^

Our study represents the most detailed analysis of blood-pressure-lowering treatment effect by age and blood pressure to date. However, our study has several limitations, which should be taken into account when interpreting our results. Our effect estimates for people aged 85 years and older at randomisation (mean age 90 years at the end of the trial) were uncertain because of the comparatively smaller number of participants developing the main outcome compared with other age groups in the study. Relatedly, because of the typically restricted eligibility criteria of RCTs, other groups such as those with a high multimorbidity and polypharmacy burden, frail individuals, and individuals living in institutions have been under-represented.^39^ The generalisability of the findings to these highly relevant and growing patient groups remains uncertain.^40^, ^41^, ^42^ Future studies such as ATEMPT (the Anti-hypertensive Treatment Evaluation in Multimorbid and Polymedicated patients Trial; ISRCTN17647940) shall address those limitations and investigate treatment effects on several additional patient-important outcomes. We acknowledge that clinical decisions cannot be deferred until such evidence emerges. However, in the absence of any strong evidence for excess harms from randomised studies, we believe that it is appropriate for patients who are on blood-pressure-lowering pharmacotherapy to continue receiving such treatment if well-tolerated and when prevention of fatal and non-fatal cardiovascular events remains of importance.

In conclusion, we found no evidence to substantiate the common approach of withholding antihypertensive treatment for older adults, in particular when their blood pressure is not highly elevated. Although the findings for people aged 85 years or more at study entry were less compelling, the overall patterns were consistent and suggestive of worthwhile reductions in cardiovascular outcomes across all age groups. Although clinical decision making for initiation and continuation of pharmacological blood-pressure-lowering will continue to be based on harm-benefit trade-offs for any individual, our study does not support the common belief that such trade-offs justify the overemphasis of several clinical practice guidelines on an individual's age or starting blood pressure. Therefore, pharmacological blood pressure reduction should be considered as an important treatment option for the prevention of cardiovascular events even in those aged 80 years or older and guidelines should be simplified to remove any differing blood pressure thresholds by age.

## Data sharing

The governance of the BPLTTC have been reported previously. The BPLTTC is governed by the University of Oxford's policies on research integrity and codes of practice and follows the university's policy on the management of research data and records. Scientific activities based on BPLTT dataset are overseen by the BPLTT Steering Committee. All data shared with the BPLTTC are considered confidential and will not be provided to any third party. Requests for data should be made directly to the data custodians of individual trials. Information about individual projects is posted at https://www.bplltc.org.

## Declaration of interests

MN reports grants from the British Heart Foundation, outside the submitted work. DC reports grants from the British Heart Foundation, during the conduct of the study. KR reports grants from the British Heart Foundation, the UK Research and Innovation Global Challenges Research Fund, Oxford Martin School, University of Oxford, and NIHR Oxford Biomedical Research Centre, University of Oxford, during the conduct of the study; personal fees from *Heart*, outside the submitted work; and research support and consulting fees to the University by Medtronic. JC reports grants from the National Health and Medical Research Council of Australia, outside the submitted work. CJP has recived grants from the US National Institutes of Health/National Heart, Lung and Blood Institute, BioCardia, GE Health Care, Caladrius Biosciences, Merck, Sanofi, CSL Behring, XyloCor Therapeutics, Mesoblast, Ventrix, and Athersys. All other authors declare no competing interests.
