# Supplementary material for: Age-stratified and blood-pressure-stratified effects of blood-pressure-lowering pharmacotherapy for the prevention of cardiovascular disease and death: an individual participant-level data meta-analysis
Source: Lancet. 2021 Sep 18;398(10305):1053–64. doi: 10.1016/S0140-6736(21)01921-8 (PMC8473559; doi:10.1016/S0140-6736(21)01921-8)
Supplement: Supplementary appendix [file mmc1.pdf]

THE LANCET

Supplementary appendix

This appendix formed part of the original submission and has been peer reviewed. We post it as supplied by the authors.

Supplement to: The Blood Pressure Lowering Treatment Trialists' Collaboration. Age-stratified and blood-pressure-stratified effects of blood-pressure-lowering pharmacotherapy for the prevention of cardiovascular disease and death: an individual participant-level data meta-analysis. *Lancet* 2021; published online Aug 27. http://dx.doi.org/10.1016/S0140-6736(21)01921-8.

Supplementary Materials

Contents

Supplementary Materials 1
Table S1. A comparison of the difference in clinical guidelines regarding the age of patients. 2
Table S2. Characteristics of randomised clinical trials included in the analysis. 3
Table S3. Unstandardised effects of systolic blood pressure-lowering treatment on primary and secondary outcomes, stratified by age categories and trial design. 7
Figure S1. Effects of diastolic blood pressure-lowering treatment on primary and secondary outcomes, stratified by age categories. 8
Figure S2. Age-specific relative effects of blood pressure-lowering treatment on all-cause death, by systolic blood pressure categories at baseline. 9
Figure S3. Age-specific relative effects of blood pressure-lowering treatment on all-cause death, by diastolic blood pressure categories at baseline. 10
Figure S4. The unstandardised effects of blood pressure-lowering treatment on risk of primary and secondary outcomes stratified by age categories. 11
References 12

Table S1. A comparison of the difference in clinical guidelines regarding the age of patients.

	ACC/AHA 2017	ACP/AAFP 2017	ESC/ESH 2018	ISH 2020	NICE 2019
**Definition of older patients**	≥65 years	≥60 years	Elderly 65-79 years<br>Very old ≥80 years	≥65 years	Over 80 years
**BP threshold for initiation of pharmacotherapy**	≥130/80 mmHg	SBP ≥150 mmHg	Elderly ≥140/90 mmHg<br>Very old ≥160/90 mmHg	≥140/90 mmHg	>150/90 mmHg
**Blood pressure target**	<130/80 mmHg	SBP <150 mmHg	SBP 130-139 mmHg<br>DBP 70-79mmHg	<140/90 mmHg	No target

BP: blood pressure, SBP: systolic blood pressure, DBP: diastolic blood pressure, ACC/AHA: the American College of Cardiology/American Heart Association, ACP: The American College of Physicians, AAFP: American Academy of Family Physicians, ESC/ESH: European Society of Cardiology/ European Society of Hypertension, ISH: International Society of Hypertension. NICE: The National Institute for Health and Care Excellence, England

Table S2. Characteristics of randomised clinical trials included in the analysis.

Trial	Type of trial	Setting	Follow-up duration (years)	Age groups					Intervention	Comparator	Definition of Primary outcome	DBP difference (mmHg) excluding first 12 months	SBP difference (mmHg) excluding first 12 months	Inclusion criteria	Exclusion criteria
				Aged less than 55 years	Aged 55 to 64 years	Aged 65 to 74 years	Aged 75 to 85 years	Aged 85 years or higher	No. of participants	No. of participants					
**AASK**[1]	Intensive	USA	4.8	524	356	214	0	0	More intensive (540)	Less intensive (554)	MI, Stroke, HF, CVD death	7.9	13.0	Age 18-70 years, African-American, hypertension, renal disease (GFR=20-65 ml/min per $1.73m^2$)	DBP <95 mmHg, diabetes, urine protein:creatinine ratio >25, recent malignant or hypertension, non-blood pressure-related CKD, serious systemic disease, heart failure
**ABCD**[2]	Intensive	USA	4.7	337	365	246	2	0	More intensive (474)	Less intensive (476)	MI or IHD, Stroke, HF, CVD death	6.9	7.7	Age 0-74 years, with T2D, DBP ≥80 mmHg, not on antihypertensive treatment	Recent CAD or CeVD, heart failure, renal disease
**ACCORD**[3]	Intensive	USA and Canada	4.7	291	2809	1379	254	0	More intensive (2362)	Less intensive (2371)	IHD or non-fatal MI, Stroke, HF, CVD death	1.8	13.9	Age ≥40y years with CVD or ≥50 years with substantial atherosclerosis, T2D, HbA1c ≥7.5%, albuminuria, LVH or ≥2 CVD risk factors (dyslipidaemia, hypertension, smoking, obesity); SBP 130-180 mmHg and taking ≤3 antihypertensive drugs, 24-hour protein excretion rate <1g	Body mass index ≥45 kg/m^2, serum creatinine ≥132.6 μmol/l and other serious illness
**ACTIVE I** [4]	Placebo-controlled	Multi-country	4.1	457	1294	2301	1858	224	ARB (3058)	Placebo (3076)	MI, Stroke, HF, CVD death	1.4	2.6	Atrial fibrillation, ≥1 risk factor (age ≥75 years, on antihypertensive treatment, history of stroke, TIA or non-CNS embolism, LVEF <45%, PVD, or age 55-74 years with either CAD or diabetes)	Use of anticoagulant, peptic ulcer disease in past 6 months, history of intracerebral haemorrhage, thrombocytopaenia or mitral stenosis
**ADVANCE**[5]	Placebo-controlled	Multi-country	4.2	17	4510	5605	995	13	ACEI and Diuretic (5569)	Placebo (5571)	MI, Stroke, HF, CVD death	2.1	5.4	Age ≥55 years T2D (diagnosed aged ≥30y), ≥1 major CVD or ≥1 CVD risk factor (microvascular disease, smoking, dyslipidaemia, microalbuminuria, T2D for ≥10 years, age ≥65 years)	HbA1c target (≤6.5%), definite indication for long-term insulin therapy
**ALLHAT**[6]	Drug classes comparison	Multi-country	4.8	0	18088	17046	6451	833	Diuretic (15255)	ACEI, CCB and Alpha-blockers (27163)	MI or IHD, Stroke, HF, CVD death	0.1	2.0	Age ≥55y years stage 1 or 2 hypertension plus ≥1 risk factor (MI or stroke >6 months previously, left ventricular hypertrophy, T2D, smoking, HDL <0.91 mmol/l), other atherosclerotic CVD	Symptomatic or hospitalisation for heart failure, LVEF <35%
**ANBP**[7]	Placebo-controlled	Australia	3.6	2289	964	174	0	0	Diuretic (1721)	Placebo (1706)	MI or IHD, Stroke, HF, CVD death	3.8	7.5	Age 30-69 years with mild hypertension (DBP 95-110 mmHg and SBP <200 mmHg)	Antihypertensive treatment in past 3 months, recent angina or MI, stroke, hormone therapy, asthma, diabetes, gout, serious disease, tricyclic antidepressant use
**ANBP2**[8]	Drug classes comparison	Australia	4.1	0	0	4094	1979	10	Diuretic (3039)	ACEI (3044)	MI or IHD, Stroke, HF, CVD death	0.0	0.9	Age 65-84 years, SBP ≥160 mmHg or DBP ≥90 mmHg (if SBP≥140 mmHg), no recent CVD	Serious illness, plasma creatinine >221 μmol/l, malignant hypertension, dementia
**ASCOT-BPLA**[9]	Drug classes comparison	Multi-country	5.3	3070	8050	6562	1575	0	CCB-based (9639)	Beta-blocker based (9618)	MI or IHD, Stroke, HF, CVD death	2.0	2.2	Age 40-79 years, untreated (SBP ≥160 or DBP ≥100 mmHg) or treated hypertension (SBP ≥140 or DBP ≥90 mmHg), ≥3 CVD risk factors (documented LVH, abnormal ECG, T2D, PAD, previous stroke or TIA, male sex, age ≥55 years, microalbuminuria or proteinuria, smoking, TC:HDL ≥6, family history of premature coronary heart disease	Previous MI, current treatment for angina, recent CeVD, fasting triglycerides >4.5 mmol/l, heart failure, arrhythmia, haematological or biochemical abnormality at screening
**BENEDICT**[10]	Placebo-controlled	Italy	3.1	211	511	412	74	1	ACEI, CCB and ACEI/CCB (904)	Placebo (300)	MI or IHD, Stroke, HF, CVD death	1.3	1.3	Age ≥40 years, untreated SBP ≥130 / DBP ≥85 mmHg or needing treatment to attain below these levels, T2D for <25 years, urinary albumin excretion rate <20 μg/min, serum creatinine ≤133 μmol/l	HbA1c ≥11%, nondiabetic renal disease
**CAMELOT**[11]	Placebo-controlled	Multi-country	1.6	801	662	461	73	0	CCB and ACEI (1340)	Placebo (657)	MI, non-fatal Stroke, HF, CVD death	3.3	5.3	Age 30-79 years, coronary artery stenosis >20% by angiography, DBP <100 mmHg	Left middle coronary artery obstruction >50%, LVEF <40%, heart failure
**CAPPP**[12]	Drug classes comparison	Sweden and Finland	5.8	6378	4033	574	0	0	Beta-blocker and/or Diuretic (5493)	ACEI (5492)	MI or IHD, Stroke, CVD death	1.3	2.2	Age 25-66 years, DBP ≥100 mmHg on two occasions	Secondary hypertension, serum creatinine >150 μmol/, condition requiring β-blocker treatment
**CARDIO-SIS**[13]	Intensive	Italy	4.7	1	463	455	182	10	More intensive (558)	Less intensive (553)	MI, Stroke, HF	1.5	3.8	Age ≥55 years, SBP ≥150 mmHg, taking antihypertensive drug ≥12 weeks, ≥1 CV risk factor (smoking, dyslipidaemia, family history of premature CVD, prior TIA or stroke, established CAD or PAD	Fasting blood glucose ≥7 mmol/l, diabetes, serious conditions, renal disease, valvular heart disease, left ventricular hypertrophy, atrial fibrillation, substance misuse.
**CASE-J**[14]	Drug classes comparison	Japan	3.1	937	1310	1705	751	0	CCB (2349)	ARB (2354)	MI, Stroke, HF, CVD death	0.9	1.7	Age 20-85 years, ≥1 high-risk factor: SBP ≥180 or DBP ≥110 mmHg, T2D, history of angina pectoris, MI, stroke, TIA >6 months prior to screening, LVH, proteinuria or serum creatinine ≥1.3 mg/100 ml, peripheral artery obstruction	BP ≥200/120 mmHg, T1D, heart failure, ejection fraction <40%, atrial fibrillation, cancer
**COLM**[15]	Drug classes comparison	Japan	3.0	0	0	2918	2223	0	ARB and Diuretic (2573)	ARB and CCB (2568)	MI, Stroke, HF, CVD death	0.4	0.3	Age 65-84 years, hypertension (treated: BP ≥140/90 mmHg; untreated: BP ≥160/100 mmHg), CVD history or CVD risk factors (diabetes, dyslipidaemia)	Secondary/malignant hypertension, recent major CVD, revascularisation, angina pectoris

															hospitalisation or severe heart failure, atrial fibrillation, hepatic or renal dysfunction
**CONVINCE**[16]	Drug classes comparison	Multi-country	2.8	0	7994	6277	2019	186	CCB (8179)	Beta-blocker or Diuretic (8297)	MI or IHD, Stroke, HF, CVD death	0.7	0.0	Age ≥55 years, hypertension, ≥1 CVD risk factor (e.g., diabetes, smoking)	Heart failure, dysrhythmia, secondary hypertension, recent MI or stroke, renal disease, other serious disease, BP ≥190/110 mmHg without treatment
**COPE**[17]	Drug classes comparison	Japan	3.6	741	1006	1021	509	16	CCB/Diuretic and CCB/ Beta-blocker (2183)	CCB and ARB (1110)	MI, Stroke, HF, CVD death	0.4	0.4	Age 40-85 years, BP ≥140/90 mmHg	SBP ≥200 or DBP ≥120 mmHg, secondary hypertension, diabetes, recent CVD or revascularisation, heart failure, atrial fibrillation/flutter, hepatic or renal dysfunction, congenital or rheumatic heart disease, cancer
**DIABHYCAR**[18]	Placebo-controlled	Multi-country	3.9	603	1732	1929	577	71	ACEI (2443)	Placebo (2469)	MI, Stroke, HF, CVD death	0.4	0.9	Age ≥50 years, T2D, urinary albumin excretion ≥20 mg/l in two consecutive urine samples	Serum creatinine >150 μmol/l, use of insulin, ACEI or ARB, heart failure, recent MI, urinary tract infection
**Dutch TIA Trial**[19]	Placebo-controlled	The Netherlands	2.3	271	438	549	206	9	Beta-blocker (732)	Placebo (741)	non-fatal MI or IHD, Stroke, CVD death	2.0	3.1	TIA or non-disabling ischaemic stroke (Rankin Scale ≤3) in past 3 months	Cerebral ischaemia from identifiable causes other than arterial thrombosis or embolism
**E-COST**[20]	Drug classes comparison	Japan	3.1	433	603	620	301	52	ARB (1053)	Conventional (995)	Not available	Not available	Not available	Age 35-79 years, BP 140-180/90-110 mmHg	Diabetes, dysglycemia, secondary hypertension, recent MI or stroke, angina pectoris requiring β-blocker treatment, heart failure, left ventricular ejection fraction <40%
**ELSA**[21]	Drug classes comparison	Multi-country	3.4	1062	911	353	8	0	CCB (1177)	Beta-blocker (1157)	MI or IHD, Stroke, HF, CVD death	0.4	0.8	Age 45-79 years, BP 150-210/95-115 mmHg	Recent MI or stroke, and T2D
**EUROPA**[22]	Placebo-controlled	Multi-country (Europe)	4.2	3550	4284	3785	590	9	ACEI (6110)	Placebo (6108)	MI, Stroke, HF	2.2	4.6	Age ≥18 years, documented MI >3 months before screening, revascularisation >6 months before screening, >70% coronary obstruction	Heart failure, hypotension, uncontrolled hypertension, renal insufficiency, serum potassium >5.5 mmol/L
**EWPHE**[23]	Placebo-controlled	Multi-country	4.6	0	169	387	216	68	Diuretic (416)	Placebo (424)	MI, Stroke, HF, CVD death	9.5	22.4	Age ≥60 years, BP 160-239/90-119 mmHg	Curable causes of high BP, retinopathy, heart failure, stroke history, hepatitis/cirrhosis, gout, malignancy, diabetes requiring insulin treatment
**HDFP**[24]	Intensive	USA	7.2	6844	3100	996	0	0	More intensive (5553)	Less intensive (5387)	MI, Stroke, HF, CVD death	4.9	9.9	Ages 30-69 years, hypertension, DBP home readings and clinic readings ≥ 95 mmHg and 90 mm Hg, respectively	age outside the range of 30 to 69 years, terminal disease, and illness resulting in confinement to bed.
**HIJ-CREATE**[25]	Drug classes comparison	Japan	4.0	311	583	867	288	0	ARB (1024)	non-ARB (1025)	MI, Stroke, HF, CVD death	0.5	0.4	Age 20-80 years, CAD hospitalisation and hypertension (BP ≥140/90 mmHg or antihypertensive treatment use)	Secondary hypertension, recent AMI or CeVD, severe aortic valve stenosis, cardiomyopathy, serum creatinine >2 mg/dl, serum potassium >5 mmol/l, hepatic dysfunction, malignancy
**HOMED-BP**[26]	Intensive	Japan	4.9	0	3518	0	0	0	More intensive (1759)	Less intensive (1759)	MI, Stroke, HF, CVD death	0.9	2.0	Self-measured SBP 135-179 mmHg or DBP 85-119 mmHg, but not if DBP <65 or SBP <110 mmHg (clinic SBP <220 mmHg and DBP <125 mmHg)	None specified
**HOPE**[27]	Placebo-controlled	Multi-country	4.5	8	4147	4128	983	30	ACEI (4645)	Placebo (4652)	MI, Stroke	1.4	3.0	Age ≥55 years, CAD, stroke, PVD or diabetes, plus ≥1 risk factor (hypertension, dyslipidaemia, smoking, or documented microalbuminuria)	Heart failure, left ejection fraction <40%, using ACEI or Vitamin E, uncontrolled hypertension, nephropathy, or recent MI or stroke
**HYVET**[28]	Placebo-controlled	Multi-country	2.1	0	0	0	2794	1049	Diuretic (1933)	Placebo (1912)	MI, Stroke, HF, CVD death	5.1	13.1	Age ≥80y years, sustained SBP ≥160 mmHg	Accelerated or secondary hypertension, recent haemorrhagic stroke, heart failure, serum creatinine >150 μmol/L, serum potassium <3.5 or >5.5 mmol/L, gout, and dementia
**IDNT**[29]	Placebo-controlled	USA	2.6	442	805	462	2	0	ARB and CCB (1146)	Placebo (569)	Total mortality	2.8	2.8	Age 30-70 years, T2D, hypertension (BP ≥135/85 mmHg or taking anti-hypertensive drug), proteinuria, serum creatinine (μmol/l): 88 to 265 (women) or 106 to 265 (men)	None specified
**INSIGHT**[30]	Drug classes comparison	Multi-country	2.8	49	3002	2685	585	0	Diuretic (3164)	CCB (3157)	MI, Stroke, fatal HF, CVD death	0.9	1.1	Age 55-80 years, hypertensive (SBP ≥150 or DBP ≥95 mmHg, or SBP ≥160 mmHg), ≥1 other risk factor (TC ≥6.43 mmol/l, smoking, family history of premature MI, CAD, other CVD	None specified
**INVEST**[31]	Drug classes comparison	Multi-country	2.8	3004	6806	7036	3779	695	CCB (10648)	non-CCB (10672)	MI or IHD, Stroke, HF, CVD death	0.2	0.1	Age ≥50 years, documented CAD, essential hypertension requiring drug therapy, heart failure Class I-III[b]	Patients taking β-blocker within two weeks of randomization or for recent MI
**JMIC-B**[32]	Drug classes comparison	Japan	2.3	224	525	746	150	3	CCB (828)	ACEI (822)	MI or IHD, Stroke, HF, CVD death	1.5	2.0	Age <75 years, hypertension (BP ≥160/≥95 mmHg or both SBP ≥150 and DBP ≥90 mmHg, or antihypertensive treatment), CAD or meeting both criteria: history of >2 anginal attacks per week with stable frequency and ST-segment depression of ≥1 mm on stress test (or detection of MI with myocardial scintigraphy)	MI, unstable angina, DBP ≥120 mmHg, secondary hypertension, symptomatic CeVD, heart failure, atrial fibrillation/arrhythmias, renal or hepatic dysfunction, uncontrollable diabetes and familial hypercholesterolaemia
**LIFE**[33]	Drug classes comparison	Multi-country	4.9	83	3350	4098	1662	0	ARB (4605)	Beta-blocker (4588)	MI or IHD, Stroke, HF, CVD death	0.5	1.2	Age 55-80 years, hypertension (SBP 160-200 mmHg; DBP 95-115 mmHg), electrocardiogram signs of LVH	Secondary hypertension, recent MI or stroke, angina pectoris requiring treatment, heart failure or left ejection fraction ≤40%
**MOSES**[34]	Drug classes comparison	Germany and Austria	3.3	137	322	511	359	23	CCB (671)	ARB (681)	MI or IHD, Stroke, HF, CVD death	0.5	1.5	Hypertension requiring treatment, documented TIA, ischaemic stroke or cerebral haemorrhage	Internal carotid artery occlusion or stenosis >70%, heart failure, age >85 years, on anticoagulant for cardiac arrhythmia, high-grade aortic or mitral valve stenosis, unstable angina

**NICS-EH**[35]	Drug classes comparison	Japan	3.2	2	104	212	92	7	Diuretic (214)	CCB (215)	MI or IHD, Stroke, HF, CVD death	0.7	0.3	Age ≥60 years, SBP 160-220 mmHg and DBP <115 mmHg and no cardiovascular complications	None specified
**NORDIL**[36]	Drug classes comparison	Norway and Sweden	4.2	2833	4973	3062	3	0	Beta-blocker and/or Diuretic (5471)	CCB (5410)	MI or IHD, Stroke, CVD death	0.1	3.3	Age 50-74 years, untreated hypertension (DBP ≥100 mmHg on two occasions); if previously treated, DBP ≥100 mmHg on two consecutive visits at one week apart during run-in period and no treatment was given	Age <50 or ≥70y, bradycardia, secondary hypertension, atrial fibrillation, recent CeVD or MI, heart failure
**ONTARGET**[37]	Drug classes comparison	Multi-country	4.8	3	10822	10926	3684	185	ARB/ACEI (8502)	ACEI and ARB (17118)	MI or IHD, Stroke, HF, CVD death	1.0	1.9	CAD, PAD, CeVD or diabetes with end-organ damage	Heart failure, pericarditis, congenital heart disease, unexplained syncope , planned revascularisation <3 months of consent, uncontrolled hypertension, heart transplant, subarachnoid haemorrhage, renal artery disease, proteinuria, hepatic dysfunction, volume or sodium depletion, primary hyper-aldosteronism, hereditary fructose intolerance, other serious conditions
**PART 2**[38]	Placebo-controlled	New Zealand	4.6	141	249	222	5	0	ACEI (308)	Placebo (309)	MI or IHD, Stroke, HF, CVD death	3.6	6.5	Age ≤75 years, diagnosis (in past 5 year) of MI, documented CAD, TIA or intermittent claudication	Heart failure, serious nonvascular disease, SBP >160 mmHg, DBP >100 mm Hg, DBP <100 mmHg during pre-randomization run-in period
**PEACE**[39]	Placebo-controlled	Multi-country (USA, Puerto Rico, Canada and Italy)	4.7	1162	3042	3056	1030	0	ACEI (4158)	Placebo (4132)	non-fatal MI, non-fatal stroke, HF, CVD death	1.5	3.0	Age ≥50 years, documented CAD	Unstable angina, severe valvular heart disease, recent revascularisation, planned elective revascularisation, limited 5-year survival, serum creatinine >177 µmol/l, serum potassium >5.5 mmol/l
**PREVEND IT**[40]	Placebo-controlled	The Netherlands	3.8	542	179	128	15	0	ACEI (431)	Placebo (433)	MI, Stroke, HF, CVD death	2.8	5.6	Microalbuminuria, SBP <160/100 mmHg (no previous antihypertension treatment)	Creatinine clearance <60% of normal age-adjusted value
**PREVENT**[41]	Placebo-controlled	USA and Canada	3.0	339	262	207	17	0	CCB (417)	Placebo (408)	MI, Stroke, HF, CVD death	3.3	6.1	Age 30-80 years, documented CAD, DBP <95 mmHg, cholesterol <325 mg/dl, fasting blood glucose <200 mg/dl	Contraindication for dihydropyridines, uncontrolled hypertension, diabetes and other major illness
**PROFESS**[42]	Placebo-controlled	Multi-country	2.5	1369	7560	7191	3359	282	ARB (9873)	Placebo (9925)	MI or IHD, Stroke, HF, CVD death	3.4	3.4	Age ≥55 years with ischaemic stroke <90 days before randomization (later modified to include age 50 to 54 years or had stroke 90 to 120 days before randomisation if with ≥2 additional risk factors: diabetes, hypertension, smoker, obesity previous CVD, end-organ damage or hyperlipidaemia) and remained stable	Haemorrhagic stroke, severe disability after the qualifying stroke, contraindication to treatments
**PROGRESS**[43]	Placebo-controlled	Multi-country (Asia, Australasia, Europe)	3.9	1026	2005	2260	767	47	ACEI and/or Diuretic (3051)	Placebo (3054)	MI or IHD, Stroke, HF, CVD death	4.0	9.2	Stroke or TIA in past 5 years	Indication or contraindication for ACEI
**SHEP**[44]	Placebo-controlled	USA	5.0	0	757	2423	1390	166	Beta-blocker and Diuretic (2365)	Placebo (2371)	non-fatal MI, non-fatal Stroke, CVD death	4.2	12.8	Age ≥60 years, isolated systolic hypertension (BP 160-219/<90 mmHg, not on treatment)	Major CVD, cancer, alcoholic liver disease, renal dysfunction, competing risk of SHEP primary endpoint or presence of medical management exclusions
**SPRINT**[45]	Intensive	USA and Puerto Rico	3.0	609	3196	2904	2258	394	More intensive (4678)	Less intensive (4683)	MI or IHD, Stroke, HF, CVD death	7.7	14.9	Age ≥50y years, SBP 130-180 mmHg, increased CVD risk (clinical/subclinical CVD other than stroke, CKD excluding polycystic kidney disease and with eGFR of 20-60 ml/min/1.73m^2 body surface area, 10-year Framingham CVD risk ≥15%, age ≥75y)	Diabetes or prior stroke
**STOP Hypertension-2**[46]	Drug classes comparison	Sweden	4.5	0	0	2696	3856	62	Beta-blocker and/or Diuretic (2213)	ACEI and CCB (4401)	MI or IHD, Stroke, HF, CVD death	0.3	2.1	Aged 70-84 years, SBP ≥180 mmHg and/or DBP ≥105 mmHg	Not specified
**SYST-EUR**[47]	Placebo-controlled	Multi-country	2.6	0	1124	2554	880	137	CCB (2398)	Placebo (2297)	MI or IHD, Stroke, HF, CVD death	4.0	10.1	Age ≥60 years, sitting SBP 160-219 mmHg, sitting DBP <95 mmHg, and standing SBP ≥140 mmHg	Secondary hypertension, retinal haemorrhage/papilloedema, heart failure, dissecting aortic aneurysm, serum creatinine ≥180 µmol/l, recent severe nosebleeds, stroke or MI, dementia, disorders prohibiting standing position, severe CVD/non-CVD
**TRANSCEND**[48]	Placebo-controlled	Multi-country	4.9	2	2349	2580	945	50	ARB (2954)	Placebo (2972)	MI or IHD, Stroke, HF, CVD death	2.2	4.5	Intolerant to ACEI and with established CAD, PVD, CeVD or diabetes with end-organ damage	Heart failure, valvular/cardiac outflow tract obstruction, pericarditis, congenital heart disease, unexplained syncope, recent revascularisation, SBP >160 mmHg, heart transplantation, subarachnoid haemorrhage, significant renal stenosis, renal or hepatic dysfunction
**UKPDS**[49]	Intensive	UK	7.9	437	513	198	0	0	More intensive (758)	Less intensive (390)	MI or IHD, Stroke	1.2	11.2	Age 25-65 years, newly diagnosed diabetes, and hypertension (untreated: SBP ≥160 mmHg and/or DBP ≥90 mmHg; treated: SBP ≥150 mmHg and/or DBP ≥85 mmHg)	Ketonuria, recent MI, angina, heart failure, >1 major vascular episode, serum creatinine >15 µmol/, retinopathy, malignant hypertension, uncorrected endocrine abnormality, severe concurrent illness
**VALISH**[50]	Intensive	Japan	2.6	0	0	1233	1846	0	More intensive (1545)	Less intensive (1534)	MI, Stroke, HF, CVD death	1.8	5.0	Age ≥70 to <85 years, isolated hypertension (SBP >160 mmHg and DBP <90 mmHg)	Secondary or malignant hypertension, BP ≥200/≥90 mmHg, recent CeVD or MI, recent/planned revascularisation, heart failure,

															aortic stenosis, valvular heart disease, atrial fibrillation/flutter, serious arrhythmia, renal/liver dysfunction
**VALUE**[51]	Drug classes comparison	Multi-country	4.2	1160	4519	6633	2725	208	CCB-based (7596)	ARB-based (7649)	MI, Stroke, HF, CVD death	1.3	1.6	Age ≥50 years, hypertension, CVD, CVD risk factors (male sex, age >50 years, diabetes, current smoking, high cholesterol, LVH, proteinuria, serum creatinine 150 to 265 μmol/l)	Renal artery stenosis, recent CAD or CeVD, severe hepatic disease or chronic renal failure, heart failure, on monotherapy with β-blocker for CAD and hypertension
**VHAS**[52]	Drug classes comparison	Italy	1.7	693	676	45	0	0	Diuretic (707)	CCB (707)	MI or IHD, Stroke, HF, CVD death	1.3	1.7	Age 40-65 years, BP ≥160/95 mmHg	Secondary hypertension, recent stroke or TIA, CAD, PAD, bradycardia, arrhythmias, heart failure, renal or hepatic dysfunction, hyperuricaemia, hypokalemia, T1D, familial dyslipidemia, serious concomitant disease

CVD: Cardiovascular disease; MI: myocardial infraction; IHD: ischaemic heart disease; HF: heart failure; SD: Standard deviation; ACEI, angiotensin-converting enzyme inhibitor; ARB, angiotensin receptor blocker; BB, beta-blocker; CCB, calcium channel blocker.

Table S3. Unstandardised effects of systolic blood pressure-lowering treatment on primary and secondary outcomes, stratified by age categories and trial design.

	**Placebo-controlled trials**					**Blood pressure-lowering intensity trials**					**Drug class comparison trials**				
	Intervention		Comparator		Hazard ratio (95% CI)	Intervention		Comparator		Hazard ratio (95% CI)	Intervention		Comparator		Hazard ratio (95% CI)
	Events	Total	Events	Total		Events	Total	Events	Total		Events	Total	Events	Total	
Major cardiovascular events															
<55 years	399	6572	502	6182	0.76 (0.67 to 0.87)	671	4641	739	4365	0.84 (0.76 to 0.93)	415	10374	501	10177	0.83 (0.72 to 0.94)
55-64 years	1798	18164	2084	17914	0.86 (0.81 to 0.91)	830	7205	872	7013	0.88 (0.80 to 0.97)	3008	34261	4124	42575	0.98 (0.93 to 1.02)
65-74 years	2704	20429	2942	19716	0.91 (0.86 to 0.95)	465	3809	485	3725	0.92 (0.81 to 1.05)	4244	35294	6124	44446	0.95 (0.91 to 0.98)
75-84 years	1411	8281	1644	8400	0.89 (0.83 to 0.95)	132	2243	174	2231	0.78 (0.62 to 0.98)	2282	14197	3356	18297	0.98 (0.93 to 1.03)
≥85 years	227	1104	219	1039	1.07 (0.89 to 1.29)	23	190	35	210	0.76 (0.45 to 1.28)	209	948	328	1277	0.93 (0.78 to 1.11)
					Adjusted P for interaction = 0.15					*Adjusted P for interaction = 1*					*Adjusted P for interaction = 0.70*
					Unadjusted P for interaction = 0.03					*Unadjusted P for interaction = 0.66*					*Unadjusted P for interaction = 0.14*
Stroke															
<55 years	112	6571	165	6178	0.66 (0.52 to 0.83)	229	4643	294	4366	0.72 (0.61 to 0.87)	135	10376	181	10177	0.73 (0.59 to 0.92)
55-64 years	664	18187	806	17939	0.82 (0.74 to 0.91)	253	7209	297	7011	0.80 (0.68 to 0.95)	846	34281	1097	42583	0.97 (0.88 to 1.16)
65-74 years	1077	20461	1223	19753	0.89 (0.82 to 0.97)	146	3805	153	3722	0.90 (0.71 to 1.12)	1361	35328	1937	44478	0.92 (0.85 to 0.98)
75-84 years	602	8291	693	8393	0.90 (0.81 to 1.00)	39	2241	47	2229	0.84 (0.55 to 1.28)	864	14221	1214	18335	0.99 (0.91 to 1.08)
≥85 years	86	1101	91	1037	1.07 (0.79 to 1.43)	6	190	10	210	0.66 (0.24 to 1.81)	47	948	94	1277	0.72 (0.51 to 1.03)
					Adjusted P for interaction = 0.25					*Adjusted P for interaction = 1*					*Adjusted P for interaction = 0.30*
					Unadjusted P for interaction = 0.05					*Unadjusted P for interaction = 0.68*					*Unadjusted P for interaction = 0.06*
Ischaemic heart disease															
<55 years	226	6574	272	6180	0.80 (0.67 to 0.95)	440	4643	441	4365	0.93 (0.81 to 1.06)	249	10374	292	10177	0.86 (0.72 to 1.01)
55-64 years	647	18164	788	17909	0.83 (0.74 to 0.92)	551	7206	554	7008	0.92 (0.82 to 1.04)	1888	34279	2567	42582	1.01 (0.96 to 1.08)
65-74 years	865	20414	973	19707	0.86 (0.79 to 0.95)	292	3810	295	3724	0.95 (0.81 to 1.12)	2382	35321	3492	44471	0.97 (0.92 to 1.02)
75-84 years	358	8276	423	8394	0.89 (0.77 to 1.02)	67	2243	81	2231	0.87 (0.63 to 1.20)	1020	14219	1611	18324	0.95 (0.88 to 1.03)
≥85 years	37	1102	34	1038	1.18 (0.74 to 1.88)	8	190	10	210	1.00 (0.39 to 2.53)	94	948	146	1276	0.98 (0.76 to 1.27)
					Adjusted P for interaction = 1					*Adjusted P for interaction = 1*					*Adjusted P for interaction = 1*
					Unadjusted P for interaction = 0.54					*Unadjusted P for interaction = 0.98*					*Unadjusted P for interaction = 0.34*
Heart failure															
<55 years	37	6434	48	6039	0.74 (0.48 to 1.14)	36	4352	35	4224	1.01 (0.63 to 1.60)	31	5832	35	5592	0.93 (0.57 to 1.51)
55-64 years	248	15578	261	15236	0.95 (0.80 to 1.13)	90	6875	93	6827	0.98 (0.74 to 1.31)	453	29732	757	38121	0.88 (0.78 to 0.98)
65-74 years	463	16873	511	16281	0.89 (0.78 to 1.00)	66	3676	91	3654	0.74 (0.54 to 1.01)	842	33448	1369	42639	0.88 (0.81 to 0.96)
75-84 years	303	7033	389	7102	0.78 (0.67 to 0.91)	34	2241	55	2229	0.65 (0.42 to 1.00)	555	14182	822	18274	0.97 (0.87 to 1.08)
≥85 years	48	1002	44	940	1.00 (0.67 to 1.52)	15	190	12	210	1.44 (0.67 to 3.07)	66	948	100	1276	0.98 (0.72 to 1.33)
					Adjusted P for interaction = 1					*Adjusted P for interaction = 1*					*Adjusted P for interaction = 1*
					Unadjusted P for interaction = 0.41					*Unadjusted P for interaction = 0.23*					*Unadjusted P for interaction = 0.64*
Cardiovascular death															
<55 years	52	4788	50	4411	1.00 (0.68 to 1.48)	142	4354	177	4230	0.77 (0.61 to 0.96)	94	10376	105	10177	0.90 (0.68 to 1.18)
55-64 years	338	14052	355	13680	0.96 (0.83 to 1.12)	207	6647	240	6616	0.85 (0.70 to 1.02)	634	34287	823	42593	1.00 (0.91 to 1.12)
65-74 years	716	16430	707	15909	1.01 (0.91 to 1.12)	137	3469	137	3436	0.97 (0.77 to 1.23)	1172	35337	1619	44490	0.99 (0.92 to 1.07)
75-84 years	558	7553	645	7588	0.90 (0.80 to 1.00)	23	2155	30	2145	0.80 (0.46 to 1.38)	851	14224	1322	18336	0.95 (0.87 to 1.04)
≥85 years	139	1089	130	1019	1.15 (0.90 to 1.46)	9	183	14	207	0.76 (0.33 to 1.76)	128	948	184	1277	1.00 (0.80 to 1.26)
					Adjusted P for interaction = 1					*Adjusted P for interaction = 1*					*Adjusted P for interaction = 1*
					Unadjusted P for interaction = 0.35					*Unadjusted P for interaction = 0.70*					*Unadjusted P for interaction = 0.86*
All-cause mortality															
<55	174	6848	182	6342	0.89 (0.73 to 1.10)	327	4651	396	4377	0.76 (0.66 to 0.88)	234	10378	245	10178	0.95 (0.80 to 1.14)
55-64	1047	18758	1065	18204	0.95 (0.87 to 1.03)	505	7214	536	7018	0.87 (0.77 to 0.98)	1756	34291	2299	42598	1.01 (0.95 to 1.07)
65-74	1927	20791	1969	19933	0.94 (0.89 to 1.00)	327	3812	313	3729	0.97 (0.83 to 1.36)	3194	35340	4330	44489	1.00 (0.96 to 1.05)
75-84	1380	8310	1486	8414	0.97 (0.90 to 1.05)	94	2248	106	2235	0.91 (0.69 to 1.20)	2306	14232	3062	18336	1.07 (1.01 to 1.13)
≥85	293	1110	277	1039	1.09 (0.93 to 1.29)	26	190	43	210	0.72 (0.44 to 1.17)	317	948	453	1277	0.97 (0.84 to 1.12)
					Adjusted P for interaction = 1.00					*Adjusted P for interaction = 1*					*Adjusted P for interaction = 1*
					Unadjusted P for interaction = 0.49					*Unadjusted P for interaction = 0.21*					*Unadjusted P for interaction = 0.35*

Figure S1. Effects of diastolic blood pressure-lowering treatment on primary and secondary outcomes, stratified by age categories.

Forest plot shows the hazard ratios (HR) and 95% confidence intervals (CI) per 3 mmHg reduction in systolic and diastolic blood pressure respectively. Adjusted p interaction: adjusted for multiple testing using Hommel's method. Unadjusted p interaction: unadjusted for multiple testing.

	Intervention		Comparator		Hazard ratio and 95% CIs per 3 mmHg reduction in diastolic blood pressure	
	Events	Total	Events	Total		
**Major cardiovascular events**						
<55 years	1485	21594	1742	20731	0.81	[0.76; 0.87]
55-64 years	5636	59649	7080	67526	0.88	[0.84; 0.92]
65-74 years	7413	59560	9551	67934	0.92	[0.88; 0.96]
75-84 years	3825	24747	5174	28944	0.88	[0.83; 0.93]
≥85 years	459	2247	582	2528	0.97	[0.85; 1.10]
					Adjusted p interaction 0.05	
					Unadjusted p interaction 0.01	
**Stroke**						
<55 years	476	21599	640	20734	0.71	[0.63; 0.80]
55-64 years	1763	59704	2200	67569	0.84	[0.78; 0.90]
65-74 years	2584	59640	3313	68020	0.90	[0.84; 0.96]
75-84 years	1505	24783	1954	28989	0.89	[0.81; 0.97]
≥85 years	139	2247	195	2529	0.90	[0.72; 1.13]
					Adjusted p interaction 0.01	
					Unadjusted p interaction 0.002	
**Ischaemic heart disease**						
<55 years	914	21598	1005	20732	0.87	[0.79; 0.95]
55-64 years	3085	59674	3906	67538	0.91	[0.85; 0.97]
65-74 years	3535	59592	4758	67964	0.93	[0.87; 1.00]
75-84 years	1444	24771	2115	28977	0.86	[0.78; 0.94]
≥85 years	139	2248	190	2528	1.04	[0.80; 1.36]
					Adjusted p interaction 1.00	
					Unadjusted p interaction 0.35	
**Heart failure**						
<55 years	104	16627	118	15870	0.87	[0.67; 1.13]
55-64 years	791	52213	1111	60224	0.96	[0.85; 1.09]
65-74 years	1371	54036	1971	62627	0.89	[0.80; 0.99]
75-84 years	892	23482	1266	27625	0.77	[0.69; 0.86]
≥85 years	129	2145	156	2427	1.01	[0.79; 1.29]
					Adjusted p interaction 1.00	
					Unadjusted p interaction 0.79	
**Cardiovascular death**						
<55 years	288	19525	332	18827	0.85	[0.73; 0.99]
55-64 years	1179	55006	1418	62915	0.92	[0.84; 1.00]
65-74 years	2025	55269	2463	63884	0.95	[0.88; 1.03]
75-84 years	1432	23958	1997	28086	0.91	[0.83; 0.99]
≥85 years	276	2224	328	2505	1.04	[0.88; 1.23]
					Adjusted p interaction 1.00	
					Unadjusted p interaction 0.17	
**All-cause death**						
<55 years	735	21879	823	20903	0.85	[0.77; 0.94]
55-64 years	3308	60278	3900	67835	0.92	[0.87; 0.98]
65-74 years	5448	59959	6612	68179	0.94	[0.89; 0.99]
75-84 years	3780	24796	4654	28994	1.00	[0.95; 1.06]
≥85 years	636	2248	773	2526	0.99	[0.89; 1.11]
					Adjusted p interaction 0.05	
					Unadjusted p interaction 0.01	

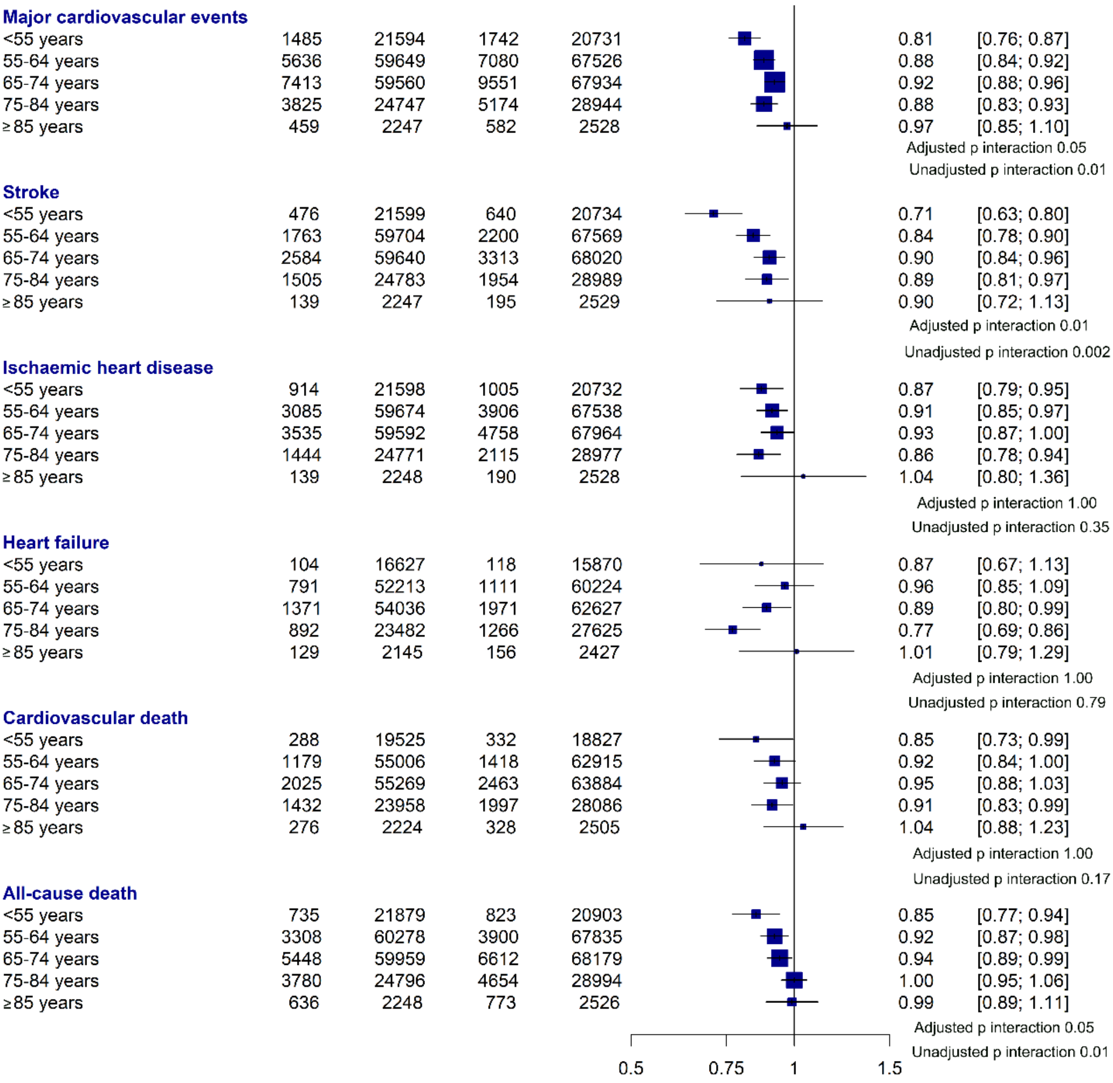

Figure S2. Age-specific relative effects of blood pressure-lowering treatment on all-cause death, by systolic blood pressure categories at baseline.

Forest plot shows the hazard ratios and 95% confidence intervals (CI) per 5 mmHg reduction in systolic blood pressure. Adjusted p interaction: adjusted for multiple testing using Hommel's method. Unadjusted p interaction: unadjusted for multiple testing

	Intervention		Comparator		Hazard ratio and 95% CIs per 5 mmHg reduction in systolic blood pressure	
	Events	Total	Events	Total		
**Aged less than 55 years**						
<120 mmHg	28	1289	38	1088	0.74	[0.44; 1.23]
120-129 mmHg	60	1956	63	1897	0.99	[0.69; 1.43]
130-139 mmHg	95	3193	95	2956	0.95	[0.69; 1.31]
140-149 mmHg	135	4337	154	4173	0.89	[0.70; 1.13]
150-159 mmHg	106	3759	137	3576	0.76	[0.58; 1.00]
160-169 mmHg	106	3379	125	3325	0.85	[0.65; 1.11]
≥170 mmHg	185	3675	197	3711	0.96	[0.77; 1.19]
					Adjusted p interaction 1.00	
					Unadjusted p interaction 0.77	
**Aged 55 - 64 years**						
<120 mmHg	193	3239	243	3893	0.92	[0.72; 1.17]
120-129 mmHg	293	5664	366	6853	1.04	[0.88; 1.23]
130-139 mmHg	447	8799	588	10376	0.92	[0.81; 1.05]
140-149 mmHg	594	11586	706	13408	1.00	[0.88; 1.13]
150-159 mmHg	585	10600	699	12139	0.98	[0.87; 1.11]
160-169 mmHg	453	9454	508	10050	0.98	[0.86; 1.11]
≥170 mmHg	651	10325	743	10829	0.91	[0.82; 1.01]
					Adjusted p interaction 1.00	
					Unadjusted p interaction 0.75	
**Aged 65 - 74 years**						
<120 mmHg	255	2482	347	2927	0.88	[0.73; 1.07]
120-129 mmHg	413	4674	544	5447	0.97	[0.82; 1.14]
130-139 mmHg	737	7500	892	8929	1.03	[0.92; 1.16]
140-149 mmHg	991	10614	1194	12595	1.01	[0.91; 1.13]
150-159 mmHg	942	10107	1166	11774	0.97	[0.86; 1.09]
160-169 mmHg	830	10973	1022	11790	0.91	[0.82; 1.00]
≥170 mmHg	1219	13272	1402	14508	0.96	[0.88; 1.04]
					Adjusted p interaction 1.00	
					Unadjusted p interaction 0.20	
**Aged 75 - 84 years**						
<120 mmHg	152	735	174	907	1.08	[0.82; 1.42]
120-129 mmHg	257	1483	336	1796	1.02	[0.84; 1.24]
130-139 mmHg	458	2656	522	3082	1.01	[0.86; 1.19]
140-149 mmHg	622	3909	738	4576	1.00	[0.86; 1.16]
150-159 mmHg	573	3610	712	4500	1.03	[0.88; 1.21]
160-169 mmHg	623	5185	766	5549	0.99	[0.89; 1.10]
≥170 mmHg	1093	7200	1403	8554	0.97	[0.90; 1.05]
					Adjusted p interaction 1.00	
					Unadjusted p interaction 0.47	
**Aged 85 years or higher**						
<120 mmHg	21	53	23	68	1.02	[0.46; 2.26]
120-129 mmHg	48	161	44	141	0.95	[0.58; 1.54]
130-139 mmHg	67	225	93	283	0.93	[0.65; 1.33]
140-149 mmHg	102	325	141	411	0.86	[0.63; 1.18]
150-159 mmHg	88	259	130	383	1.03	[0.76; 1.40]
160-169 mmHg	108	495	128	513	0.88	[0.68; 1.14]
≥170 mmHg	202	730	214	727	0.99	[0.79; 1.24]
					Adjusted p interaction 1.00	
					Unadjusted p interaction 0.84	

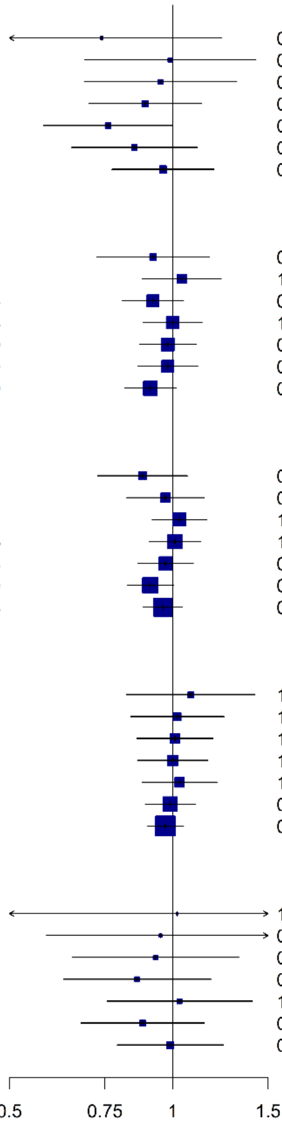

Figure S3. Age-specific relative effects of blood pressure-lowering treatment on all-cause death, by diastolic blood pressure categories at baseline.

Forest plot shows the hazard ratios and 95% confidence intervals (CI) per 3 mmHg reduction in diastolic blood pressure. Adjusted p interaction: adjusted for multiple testing using Hommel's method. Unadjusted p interaction: unadjusted for multiple testing

	Intervention		Comparator		Hazard ratio and 95% CIs per 3 mmHg reduction in diastolic blood pressure	
	Events	Total	Events	Total		
**Aged less than 55 years**						
<70 mmHg	15	497	7	443	2.28	[0.77; 6.74]
70-79 mmHg	48	1698	60	1561	0.78	[0.52; 1.16]
80-89 mmHg	88	3901	108	3599	0.70	[0.49; 1.00]
90-99 mmHg	266	7064	278	6635	0.94	[0.79; 1.12]
100-109 mmHg	186	6186	226	6135	0.82	[0.67; 1.00]
≥110 mmHg	112	2242	130	2354	0.90	[0.70; 1.16]
					Adjusted p interaction 1.00	
					Unadjusted p interaction 0.37	
**Aged 55 - 64 years**						
<70 mmHg	211	2789	246	3298	1.00	[0.75; 1.34]
70-79 mmHg	496	9034	660	10952	0.81	[0.68; 0.96]
80-89 mmHg	952	17813	1133	20605	0.98	[0.86; 1.12]
90-99 mmHg	917	17766	1061	19657	0.95	[0.86; 1.05]
100-109 mmHg	447	9578	566	10335	0.84	[0.73; 0.96]
≥110 mmHg	193	2688	187	2701	0.98	[0.80; 1.21]
					Adjusted p interaction 1.00	
					Unadjusted p interaction 0.25	
**Aged 65 - 74 years**						
<70 mmHg	478	4494	685	5262	0.79	[0.67; 0.94]
70-79 mmHg	1105	11693	1361	13903	1.07	[0.94; 1.21]
80-89 mmHg	1789	20022	2112	22853	0.95	[0.86; 1.05]
90-99 mmHg	1292	15325	1548	17147	0.97	[0.88; 1.07]
100-109 mmHg	536	6400	652	7028	0.83	[0.73; 0.95]
≥110 mmHg	184	1683	209	1772	0.93	[0.75; 1.16]
					Adjusted p interaction 0.02	
					Unadjusted p interaction 0.004	
**Aged 75 - 84 years**						
<70 mmHg	464	2694	533	3155	1.10	[0.94; 1.28]
70-79 mmHg	863	5710	1064	6486	0.95	[0.84; 1.08]
80-89 mmHg	1184	8163	1458	9564	1.03	[0.91; 1.16]
90-99 mmHg	897	5885	1076	6811	0.96	[0.87; 1.06]
100-109 mmHg	280	1875	398	2344	0.95	[0.79; 1.14]
≥110 mmHg	90	448	122	603	1.06	[0.76; 1.47]
					Adjusted p interaction 1.00	
					Unadjusted p interaction 0.38	
**Aged 85 years or higher**						
<70 mmHg	76	306	109	398	1.08	[0.80; 1.46]
70-79 mmHg	139	507	174	582	0.96	[0.74; 1.24]
80-89 mmHg	204	658	221	715	0.95	[0.72; 1.25]
90-99 mmHg	169	624	207	673	0.85	[0.69; 1.05]
100-109 mmHg	40	137	55	140	0.86	[0.57; 1.30]
≥110 mmHg	7	14	7	16	1.24	[0.38; 4.04]
					Adjusted p interaction 1.00	
					Unadjusted p interaction 0.72	

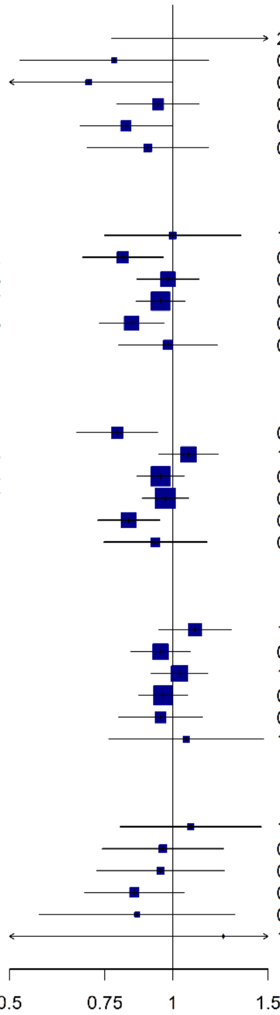

Figure S4. The unstandardised effects of blood pressure-lowering treatment on risk of primary and secondary outcomes stratified by age categories.

Forest plot shows the hazard ratios and 95% confidence intervals (CI), separately for each outcome. Adjusted p interaction: adjusted for multiple testing using Hommel's method. Unadjusted p interaction: unadjusted for multiple testing

	Intervention		Comparator		Hazard ratio and 95% CIs	
	Events	Total	Events	Total		
**Major cardiovascular events**						
<55 years	1485	21594	1742	20731	0.82	[0.77; 0.88]
55-64 years	5636	59649	7080	67526	0.93	[0.90; 0.96]
65-74 years	7413	59560	9551	67934	0.93	[0.90; 0.96]
75-84 years	3825	24747	5174	28944	0.94	[0.90; 0.98]
≥85 years	459	2247	582	2528	0.99	[0.88; 1.12]
					Adjusted p interaction 0.05	
					Unadjusted p interaction 0.01	
**Stroke**						
<55 years	476	21599	640	20734	0.71	[0.63; 0.80]
55-64 years	1763	59704	2200	67569	0.89	[0.84; 0.95]
65-74 years	2584	59640	3313	68020	0.90	[0.86; 0.95]
75-84 years	1505	24783	1954	28989	0.94	[0.88; 1.01]
≥85 years	139	2247	195	2529	0.89	[0.72; 1.11]
					Adjusted p interaction 0.01	
					Unadjusted p interaction 0.002	
**Ischaemic heart disease**						
<55 years	914	21598	1005	20732	0.87	[0.80; 0.95]
55-64 years	3085	59674	3906	67538	0.96	[0.92; 1.00]
65-74 years	3535	59592	4758	67964	0.93	[0.89; 0.97]
75-84 years	1444	24771	2115	28977	0.94	[0.88; 1.00]
≥85 years	139	2248	190	2528	1.04	[0.84; 1.29]
					Adjusted p interaction 1.00	
					Unadjusted p interaction 0.35	
**Heart failure**						
<55 years	104	16627	118	15870	0.87	[0.67; 1.13]
55-64 years	791	52213	1111	60224	0.91	[0.83; 1.00]
65-74 years	1371	54036	1971	62627	0.87	[0.81; 0.93]
75-84 years	892	23482	1266	27625	0.90	[0.82; 0.98]
≥85 years	129	2145	156	2427	1.02	[0.81; 1.28]
					Adjusted p interaction 1.00	
					Unadjusted p interaction 0.79	
**Cardiovascular death**						
<55 years	288	19525	332	18827	0.84	[0.72; 0.98]
55-64 years	1179	55006	1418	62915	0.97	[0.90; 1.05]
65-74 years	2025	55269	2463	63884	1.00	[0.94; 1.06]
75-84 years	1432	23958	1997	28086	0.93	[0.87; 0.99]
≥85 years	276	2224	328	2505	1.05	[0.89; 1.23]
					Adjusted p interaction 1.00	
					Unadjusted p interaction 0.17	
**All-cause death**						
<55 years	735	21879	823	20903	0.85	[0.77; 0.93]
55-64 years	3308	60278	3900	67835	0.98	[0.94; 1.03]
65-74 years	5448	59959	6612	68179	0.98	[0.95; 1.02]
75-84 years	3780	24796	4654	28994	1.03	[0.99; 1.08]
≥85 years	636	2248	773	2526	1.00	[0.90; 1.11]
					Adjusted p interaction 0.05	
					Unadjusted p interaction 0.01	

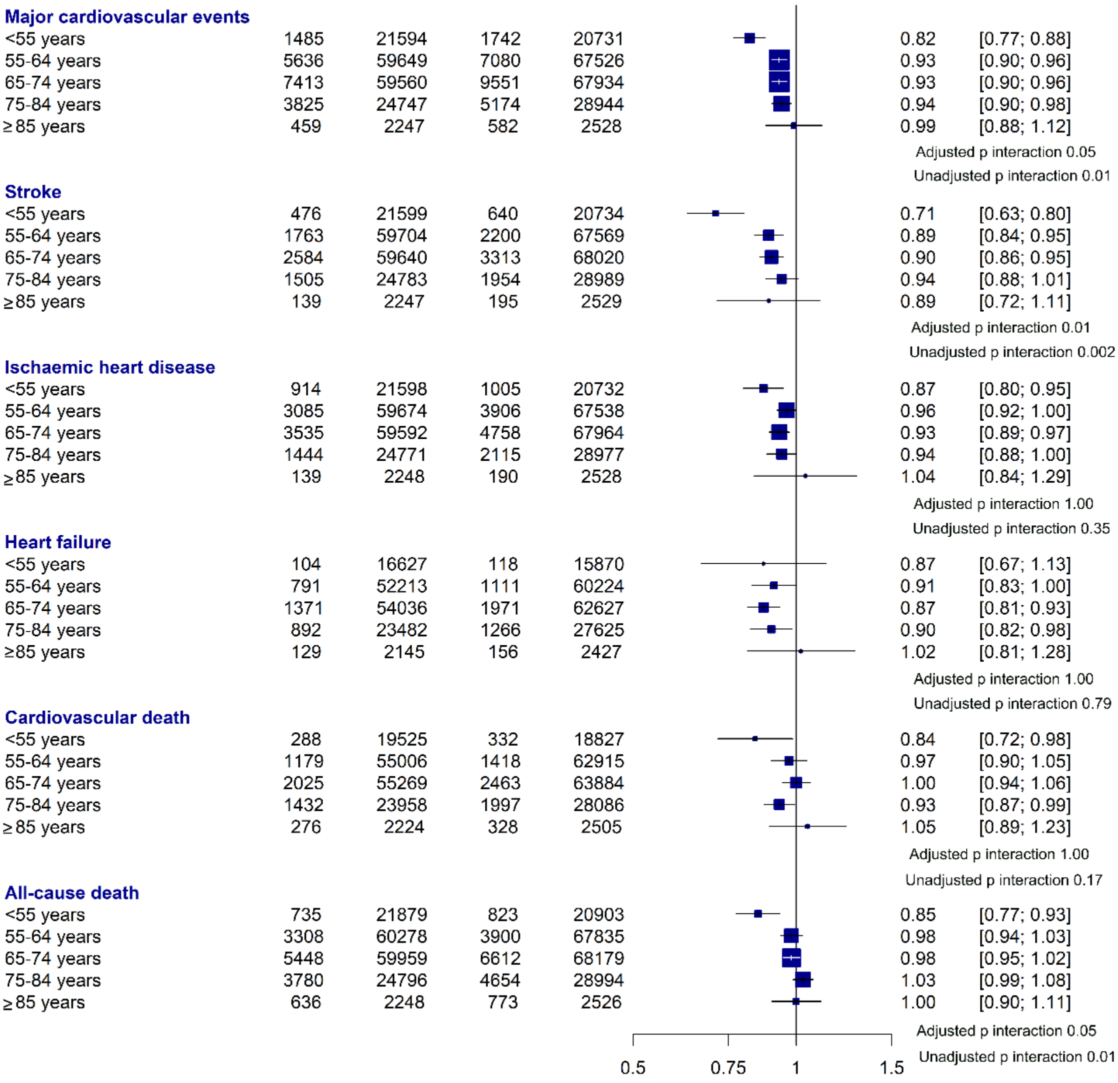

References

1 Appel LJ, Wright JT, Greene T. Intensive blood-pressure control in hypertensive chronic kidney disease. *N Engl J Med* 2010; **363**: 2565–6.

2 Schrier RW, Estacio RO, Jeffers B. Appropriate blood pressure control in NIDDM (ABCD) Trial. In: Diabetologia. 1996: 1646–54.

3 Cushman WC, Evans GW, Byington RP, *et al.* Effects of intensive blood-pressure control in type 2 diabetes mellitus. *N Engl J Med* 2010; **362**: 1575–85.

4 Yusuf S, Healey JS, Pogue J, *et al.* Irbesartan in patients with atrial fibrillation. *N Engl J Med* 2011; **364**: 928–38.

5 Patel A. Effects of a fixed combination of perindopril and indapamide on macrovascular and microvascular outcomes in patients with type 2 diabetes mellitus (the ADVANCE trial): a randomised controlled trial. *Lancet* 2007; **370**: 829–40.

6 Group TAO and C for the ACR, Coordinators TAO and, Antihypertensive T, Treatment L. Major Outcomes in High-Risk Hypertensive Patients Randomized to Angiotensin-Converting Enzyme Inhibitor or Calcium Channel Blocker vs Diuretic. *JAMA J Am Med Assoc* 2002; **288**: 2981–97.

7 Doyle AE. The Australian National blood pressure study. *Trends Pharmacol Sci* 1981; **2**: 293–6.

8 Wing LMH, Reid CM, Ryan P, *et al.* A comparison of outcomes with angiotensin-converting-enzyme inhibitors and diuretics for hypertension in the elderly. *N Engl J Med* 2003; **348**: 583–92.

9 Dahlöf B, Sever PS, Poulter NR, *et al.* Prevention of cardiovascular events with an antihypertensive regimen of amlodipine adding perindopril as required versus atenolol adding bendroflumethiazide as required, in the Anglo-Scandinavian Cardiac Outcomes Trial-Blood Pressure Lowering Arm (ASCOT-B. *Lancet* 2005; **366**: 895–906.

10 Ruggenenti P, Fassi A, Ilieva AP, *et al.* Preventing microalbuminuria in type 2 diabetes. *N Engl J Med* 2004; **351**: 1941–51.

11 Park S, Yan P, Cerezo C, Jeffers BW. Effect of visit-to-visit blood pressure variability on cardiovascular events in patients with coronary artery disease and well-controlled blood pressure. *J Am Soc Hypertens* 2016; **10**: 799–810.

12 Hansson L, Lindholm LH, Niskanen L, *et al.* Effect of angiotensin-converting-enzyme inhibition compared with conventional therapy on cardiovascular morbidity and mortality in hypertension: the Captopril Prevention Project (CAPPP) randomised trial. *Lancet* 1999; **353**: 611–6.

13 Verdecchia P, Staessen JA, Angeli F, *et al.* Usual versus tight control of systolic blood pressure in non-diabetic patients with hypertension (Cardio-Sis): an open-label randomised trial. *Lancet* 2009; **374**: 525–33.

14 Nakao K, Hirata M, Oba K, *et al.* Role of diabetes and obesity in outcomes of the candesartan antihypertensive survival evaluation in Japan (CASE-J) trial. *Hypertens Res* 2010; **33**: 600–6.

15 Ogihara T, Saruta T, Rakugi H, *et al.* Combinations of olmesartan and a calciumchannel blocker or a diuretic inelderly hypertensive patients: A randomized, controlled trial. *J Hypertens* 2014; **32**: 2054–63.

16 Black HR, Elliott WJ, Grandits G, *et al.* Principal Results of the Controlled Onset Verapamil

Investigation of Cardiovascular End Points (CONVINCE) Trial. *J Am Med Assoc* 2003; **289**: 2073–82.

17 Matsuzaki M, Ogihara T, Umemoto S, *et al.* Prevention of cardiovascular events with calcium channel blocker-based combination therapies in patients with hypertension: A randomized controlled trial. *J Hypertens* 2011; **29**: 1649–59.

18 Marre M, Lievre M, Chatellier G, Mann JFE, Passa P, Ménard J. Effects of low dose ramipril on cardiovascular and renal outcomes in patients with type 2 diabetes and raised excretion of urinary albumin: Randomised, double blind, placebo controlled trial (the DIABHYCAR study). *Br Med J* 2004; **328**: 495–9.

19 Koudstaal PJ, Algra A, Pop GA, Kappelle LJ, van Latum JC, van Gijn J. Risk of cardiac events in atypical transient ischaemic attack or minor stroke. The Dutch TIA Study Group. *Lancet (London, England)* 1992; **340**: 630–3.

20 Suzuki H, Kanno Y, Kanai A, *et al.* Effects of candesartan on cardiovascular outcomes in Japanese hypertensive patients. *Hypertens Res* 2005; **28**: 307–14.

21 Zanchetti A, Bond MG, Hennig M, *et al.* Calcium antagonist lacidipine slows down progression of asymptomatic carotid atherosclerosis: Principal results of the European Lacidipine Study on Atherosclerosis (ELSA), a randomized, double-blind, long-term trial. *Circulation* 2002; **106**: 2422–7.

22 Fox KM, Bertrand M, Ferrari R, *et al.* Efficacy of perindopril in reduction of cardiovascular events among patients with stable coronary artery disease: Randomised, double-blind, placebo-controlled, multicentre trial (the EUROPA study). *Lancet* 2003; **362**: 782–8.

23 Amery A, Brixko P, Clement D, *et al.* MORTALITY AND MORBIDITY RESULTS FROM THE EUROPEAN WORKING PARTY ON HIGH BLOOD PRESSURE IN THE ELDERLY TRIAL. *Lancet* 1985; **325**: 1349–54.

24 Five-Year Findings of the Hypertension Detection and Follow-up Program: I. Reduction in Mortality of Persons With High Blood Pressure, Including Mild Hypertension. *JAMA J Am Med Assoc* 1979; **242**: 2562–71.

25 Kasanuki H, Hagiwara N, Hosoda S, *et al.* Angiotensin II receptor blocker-based vs. non-angiotensin II receptor blocker-based therapy in patients with angiographically documented coronary artery disease and hypertension: The Heart Institute of Japan Candesartan Randomized Trial for Evaluation in. *Eur Heart J* 2009; **30**: 1203–12.

26 Asayama K, Ohkubo T, Metoki H, *et al.* Cardiovascular outcomes in the first trial of antihypertensive therapy guided by self-measured home blood pressure. *Hypertens Res* 2012; **35**: 1102–10.

27 Sharma AM, Pischon T, Engeli S. Effect of ramipril on cardiovascular events in high-risk patients. *N Engl J Med* 2000; **343**.

28 Beckett NS, Peters R, Fletcher AE, *et al.* Treatment of hypertension in patients 80 years of age or older. *N Engl J Med* 2008; **358**: 1887–98.

29 Lewis EJ, Hunsicker LG, Clarke WR, *et al.* Renoprotective effect of the angiotensin-receptor antagonist irbesartan in patients with nephropathy due to type 2 diabetes. *N Engl J Med* 2001; **345**: 851–60.

30 Brown MJ, Palmer CR, Castaigne A, *et al.* Morbidity and mortality in patients randomised to double-blind treatment with a long-acting calcium-channel blocker or diuretic in the

International Nifedipine GITS study: Intervention as a Goal in Hypertension Treatment (INSIGHT). *Lancet* 2000; **356**: 366–72.

31 Pepine CJ, Handberg EM, Cooper-DeHoff RM, *et al.* A Calcium Antagonist vs a Non-Calcium Antagonist Hypertension Treatment Strategy for Patients with Coronary Artery Disease the International Verapamil-Trandolapril Study (INVEST): A Randomized Controlled Trial. *J Am Med Assoc* 2003; **290**: 2805–16.

32 Yui Y, Sumiyoshi T, Kodama K, *et al.* Comparison of nifedipine retard with angiotensin converting enzyme inhibitors in Japanese hypertensive patients with coronary artery disease: The Japan Multicenter Investigation for Cardiovascular Diseases-B (JMIC-B) randomized trial. *Hypertens Res* 2004; **27**: 181–91.

33 Lindholm LH, Ibsen H, Dahlöf B, *et al.* Cardiovascular morbidity and mortality in patients with diabetes in the Losartan Intervention For Endpoint reduction in hypertension study (LIFE): A randomised trial against atenolol. *Lancet* 2002; **359**: 1004–10.

34 Schrader J, Lüders S, Kulschewski A, *et al.* Morbidity and mortality after stroke, eprosartan compared with nitrendipine for secondary prevention: Principal results of a prospective randomized controlled study (MOSES). *Stroke* 2005; **36**: 1218–24.

35 Kuramoto K. Randomized double-blind comparison of a calcium antagonist and a diuretic in elderly hypertensives: National intervention cooperative study in elderly hypertensives study group. *Hypertension* 1999; **34**: 1129–33.

36 Hansson L, Hedner T, Lund-Johansen P, *et al.* Randomised trial of effects of calcium antagonists compared with diuretics and β-blockers on cardiovascular morbidity and mortality in hypertension: The Nordic Diltiazem (NORDIL) study. *Lancet* 2000; **356**: 359–65.

37 Yusuf S, Teo KK, Pogue J, *et al.* Telmisartan, ramipril, or both in patients at high risk for vascular events. *N Engl J Med* 2008; **358**: 1547–59.

38 MacMahon S, Sharpe N, Gamble G, *et al.* Randomized, placebo-controlled trial of the angiotensin-converting enzyme inhibitor, ramipril, in patients with coronary or other occlusive arterial disease. *J Am Coll Cardiol* 2000; **36**: 438–43.

39 Braunwald E, Domanski MJ, Fowler SE, *et al.* Angiotensin-converting-enzyme inhibition in stable coronary artery disease. *N Engl J Med* 2004; **351**. DOI:10.1056/NEJMoa042739.

40 Asselbergs FW, Diercks GFH, Hillege HL, *et al.* Effects of fosinopril and pravastatin on cardiovascular events in subjects with microalbuminuria. *Circulation* 2004; **110**: 2809–16.

41 Eleuteri E. Effect of amlodipine on the progression of atherosclerosis and the occurrence of clinical events. *Ital Heart J Suppl* 2001; **2**: 85–6.

42 Sacco RL, Diener HC, Yusuf S, *et al.* Aspirin and extended-release dipyridamole versus clopidogrel for recurrent stroke. *N Engl J Med* 2008; **359**: 1238–51.

43 MacMahon S, Neal B, Tzourio C, *et al.* Randomised trial of a perindopril-based blood-pressure-lowering regimen among 6105 individuals with previous stroke or transient ischaemic attack. *Lancet* 2001; **358**: 1033–41.

44 Ogihara T, Nakao K, Fukui T, *et al.* Effects of candesartan compared with amlodipine in hypertensive patients with high cardiovascular risks: Candesartan antihypertensive survival evaluation in Japan trial. *Hypertension* 2008; **51**: 393–8.

45 The SPRINT Research Group. A Randomized Trial of Intensive versus Standard Blood-Pressure Control. *N Engl J Med* 2015; **373**: 2103–16.

46 Hansson L, Lindholm LH, Ekbom T, *et al.* Randomised trial of old and new antihypertensive drugs in elderly patients: Cardiovascular mortality and morbidity the Swedish trial in old patients with hypertension-2 study. *Lancet* 1999; **354**: 1751–6.

47 JA S, R F, L T, *et al.* Randomised double-blind comparison of placebo and active treatment for older patients with isolated systolic hypertension. The Systolic Hypertension in Europe (Syst-Eur) Trial Investigators. *Lancet (London, England)* 1997; **350**: 757–64.

48 Telmisartan T, Assessment R. Effects of the angiotensin-receptor blocker telmisartan on cardiovascular events in high-risk patients intolerant to angiotensin-converting enzyme inhibitors: a randomised controlled trial. *Lancet* 2008; **372**: 1174–83.

49 Tight blood pressure control and risk of macrovascular and microvascular complications in type 2 diabetes: UKPDS 38. UK Prospective Diabetes Study Group. *BMJ* 1998; **317**: 703–13.

50 Ogihara T, Saruta T, Rakugi H, *et al.* Target blood pressure for treatment of isolated systolic hypertension in the elderly: Valsartan in elderly isolated systolic hypertension study. *Hypertension* 2010; **56**: 196–202.

51 Julius S, Kjeldsen SE, Weber M, *et al.* Outcomes in hypertensive patients at high cardiovascular risk treated with regimens based on valsartan or amlodipine: The VALUE randomised trial. *Lancet* 2004; **363**: 2022–31.

52 Zanchetti A, Agabiti Rosei E, Dal Palù C, Leonetti G, Magnani B, Pessina A. The Verapamil in Hypertension and Atherosclerosis Study (VHAS): Results of long-term randomized treatment with either verapamil or chlorthalidone on carotid intima-media thickness. *J Hypertens* 1998; **16**: 1667–76.
